# Prolonged abstinence from cocaine or morphine disrupts separable valuations during decision conflict

**DOI:** 10.1038/s41467-018-04967-2

**Published:** 2018-06-28

**Authors:** Brian M. Sweis, A. David Redish, Mark J. Thomas

**Affiliations:** 1 Graduate Program in Neuroscience & Medical Scientist Training Program, University of Minnesota, Minneapolis, MN 55455 USA; 2 Department of Neuroscience, University of Minnesota, Minneapolis, MN 55455 USA; 3 Department of Psychology, University of Minnesota, Minneapolis, MN 55455 USA

## Abstract

Neuroeconomic theories propose changes in decision making drive relapse in recovering drug addicts, resulting in continued drug use despite stated wishes not to. Such conflict is thought to arise from multiple valuation systems dependent on separable neural components, yet many neurobiology of addiction studies employ only simple tests of value. Here, we tested in mice how prolonged abstinence from different drugs affects behavior in a neuroeconomic foraging task that reveals multiple tests of value. Abstinence from repeated cocaine and morphine disrupts separable decision-making processes. Cocaine alters deliberation-like behavior prior to choosing a preferred though economically unfavorable offer, while morphine disrupts re-evaluations after rapid initial decisions. These findings suggest that different drugs have long-lasting effects precipitating distinct decision-making vulnerabilities. Our approach can guide future refinement of decision-making behavioral paradigms and highlights how grossly similar behavioral maladaptations may mask multiple underlying, parallel, and dissociable processes that treatments for addiction could potentially target.

## Abstract (web summary)

Neuroeconomic theories suggest that conflict during decision, such as exhibited by relapsing drug addicts who continue drug use despite stated wishes not to, might arise from separable processes in decision making. Here the authors test mice in a foraging task designed to separate these processes and find that mice show alterations in separable components of decision conflict following abstinence from cocaine versus morphine.

## Introduction

Cocaine and morphine can both lead to rewiring of neural circuits involved in motivated behavior^1,2^. Although these drugs have different immediate mechanisms of action, theories have suggested that they ultimately converge on a final common dysfunction in mesolimbic dopamine leading to maladaptive reinforcement learning^3,6–10^. However, it has also been hypothesized that malfunctions in decision-making systems with distinct neural circuits are capable of giving rise to multiple addiction etiologies, and that cocaine and morphine may access different malfunctions in those circuits despite producing grossly similar changes in maladaptive goal-oriented behavior^2^. So far, it has not been possible to dissect apart such changes behaviorally^11^.

**Fig. 1:**
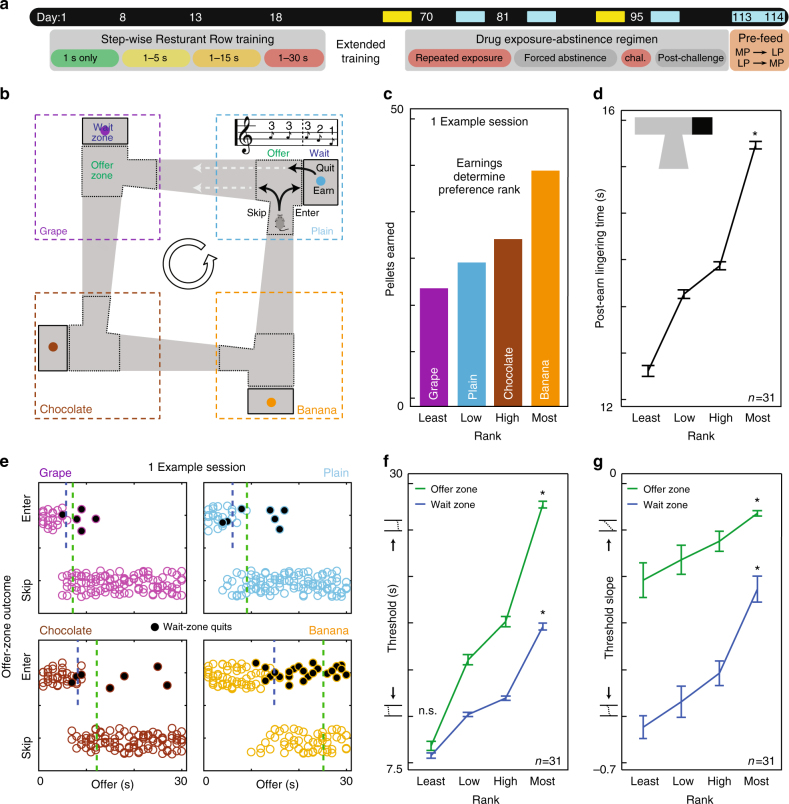
Multiple valuations in Restaurant Row. **a** Experimental timeline. Timepoints of interest marked in yellow: well-trained at baseline (days 66–70, Figs. 1 and 2); after prolonged abstinence from repeated drug exposure (days 90–94, Fig. 3). Supplementary timepoints are marked in cyan. **b** Mice were trained to run counter-clockwise around a square maze encountering serial offers for flavored rewards in four restaurants. Tone pitch indicated delay length that sounded in the offer zone, but did not countdown until after entering the wait zone. **c** Flavors were ranked from least- to most-preferred by end-of-session earnings each day. Panel shows one example session. Mice showed individual preferences that were stable across days (Supplementary Fig. 2). **d** Kruskal–Wallis (KW) tests revealed mice spent more time lingering at the reward site after earning rewards in more-preferred restaurants before moving on to the next trial (*P < 0.0001). **e** Mice entered low delays and skipped high delays in the offer zone, while infrequently quitting once in the wait zone (black dots). Dashed vertical lines represent calculated offer-zone and wait-zone thresholds of willingness to budget time. Green line indicates offer-zone threshold (all offers). Blue line indicates wait-zone threshold (entered offers). **f** KW tests revealed thresholds to enter (offer zone) and earn (wait zone) rewards were higher in more-preferred restaurants (*P < 0.0001). Post-hoc Dunn’s tests controlled for multiple comparisons revealed disparity between offer- and wait-zone thresholds was greater in more-preferred restaurants (*P < 0.0001), generating more enter-then-quit events (least-preferred, not significant, n.s., *P* > 0.05). **g** Slope of threshold fits were higher in the offer zone than wait zone and in more-preferred restaurants (KW test, *P < 0.0001). Error bars. ± 1 s.e.m. *N* = 31

We developed a neuroeconomic task in mice that reveals multiple parallel valuation algorithms and separates decision-making processes of reward conflict into behaviorally deconstructed stages^12^. Food-restricted mice traversed a square maze with four feeding sites (restaurants), each providing a different flavor, with two distinct zones: an offer zone and a wait zone (Fig. 1b, Methods). Tones sounded upon offer zone entry, whose pitch indicated a delay (pseudo-random, 1–30 s) that mice would have to wait if they chose to enter the wait zone in order to receive food reward. Mice could choose to quit during delay countdowns. Importantly, mice had 1 h to forage for their food for the day. Using different flavors instead of pellet number allowed us to measure subjective preferences (Fig. 1c) without introducing differences in time required for food consumption.

The economic key to foraging is the division of time. Time spent choosing in the offer zone, waiting in the wait zone, and remaining at the reward site after receiving food all detracts from time spent making other decisions elsewhere. Critically, choices in each of these three decision modalities (skip vs. enter, quit vs. continue to wait, leave vs. linger) are computationally distinct valuation processes that reflect economic conflict.

We find that repeated exposure to cocaine or morphine produced lasting disruptions in judgments during these instances of economic conflict. Cocaine-abstinent mice displayed impairments in deliberative valuation processes in the offer zone before ultimately accepting economically disadvantageous reward offers. Morphine-abstinent mice displayed impairments in foraging re-evaluative processes in the wait zone when correcting poor snap judgements. Together, these data demonstrate how drugs of abuse can give rise to lasting dysfunctions in fundamentally distinct decision-making valuation algorithms and suggest that individualized treatments tailored to computation-specific processes might ameliorate heterogeneous addiction subtypes.

## Results

### Separating stages of economic subjective valuations

Mice spent the majority of time lingering at the reward site after earning and consuming a reward (Supplementary Fig. 1). Interestingly, mice lingered longer in more-preferred restaurants (Fig. 1d). This decision to linger rather than leave, where no overt reward is being sought out, may represent a conditioned-place-preference-like effect^13^ associated with each restaurant’s context.

**Fig. 2:**
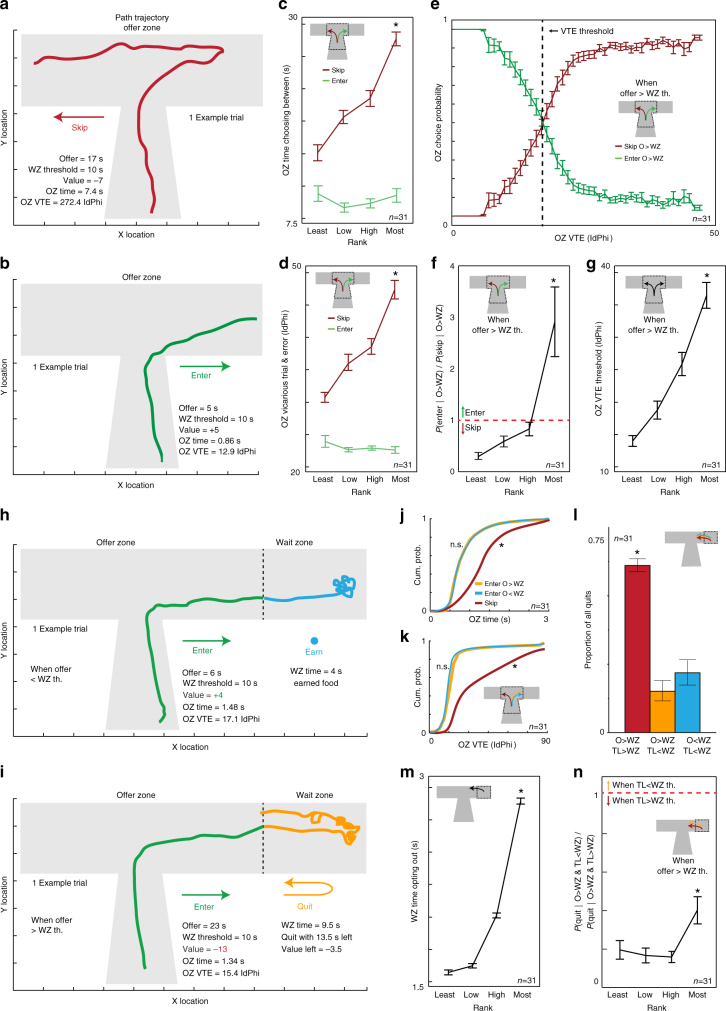
Characterizing deliberation and foraging behaviors. **a**, **b** Example of X–Y locations of a mouse’s path trajectory in the offer zone over time during a single trial. **a** Skip decision for a high delay offer. The mouse initially oriented toward entering (right) then ultimately re-oriented to skip (left). Wait-zone threshold minus offer captures the relative subjective value of the offer. Negative value denotes an economically unfavorable offer. **b** Enter decision for positively valued offer; rapid without re-orientations. Reaction time (**c**) and VTE (**d**) behavior was higher for skip compared to enter decisions and only increased in more-preferred restaurants for skip decisions (KW tests, *P < 0.0001). **e** Mice were more likely to skip negatively valued offers the more they displayed VTE behavior. Vertical dashed line indicates the amount of VTE required to skip these offers 50% of the time. **f** Mice were more likely to enter these offers in higher-preferred restaurants, entering more than skipping in only the most-preferred restaurant (KW and Sign tests, *P < 0.0001). **g** Amount of VTE required to reliably skip these offers was higher in more-preferred restaurants (KW tests, *P < 0.0001). **h**, **i** Example of path trajectory in the offer and wait zones. **h** Rapidly entering then earning a positively valued offer. **i** Rapidly entering then quitting a negatively valued offer. **j**, **k** Cumulative probability distribution of offer zone time (**j**) and VTE (**k**) for skips and enters split by offer value. Both types of enter decisions were rapid compared to skips (Kolmogorov–Smirnov (KS) tests, *P < 0.05) and indistinguishable from each other (KS tests, not significant, n.s., *P* > 0.05). **l**, **m** Majority of quits took place for negatively valued offers and while time left was still greater than wait zone thresholds (**l**), despite taking longer to quit in more-preferred restaurants (**m**, KW-D tests, *P < 0.0001). **n** Although mice were more likely to quit negatively valued offers while the amount of time left was still above wait zone thresholds in all restaurants, they were less capable of doing so in more-preferred restaurants (KW and Sign tests, *P < 0.0001). Error bars. ± 1 s.e.m. *N* = 31

We calculated offer zone thresholds of willingness to enter as a function of offered delay (Fig. 1e, Supplementary Fig. 2), and found higher thresholds in more-preferred restaurants compared to less-preferred restaurants (Fig. 1e, f). Interestingly, mice took longer in the offer zone deciding to skip than deciding to enter (Fig. 2a–c). Furthermore, decision time took longer when skipping more-preferred restaurants (Fig. 2c). These data suggest that highly desired rewards were more difficult to turn down.

Degree of adherence to thresholds can be measured via slope of fitted sigmoid functions. Steeper (more negative) slopes indicate low likelihoods of threshold violation (e.g., enter above or skip below offer zone thresholds). Threshold slope was less steep in more-preferred restaurants (Fig. 1g), suggesting highly desired reward offers blurred subjective policies to make economically advantageous judgments to skip vs. enter.

We carried out similar analyses in the wait zone for quit decisions. Wait-zone thresholds also increased for more-preferred flavors (Fig. 1e, f). However, wait-zone threshold slope was steeper than offer-zone threshold slope (Fig. 1g), indicating mice were less likely to violate wait-zone thresholds. This meant that wait-zone metrics captured a fundamentally different valuation process than the offer zone: we found no relationship between the two types of thresholds or with lingering time after accounting for ordinal ranking of flavor, even though all three valuation parameters, importantly, agreed on the ordinal ranking of a given flavor (Figs 1d, f, g, Supplementary Fig. 3).

### Approach behaviors and economic efficiency of decisions

Disparity between offer- and wait-zone thresholds was greatest (offer zone > wait zone) in more-preferred restaurants (Fig. 1f). In these restaurants, then, mice were more likely to accept offers with a higher cost than subjective value indicated that they should (Fig. 2f). This scenario—entering offers that are greater than wait-zone thresholds—is an explicit economic failure to choose a better alternative over a tantalizing reward offer. In such instances, it would have been economically advantageous to choose to skip in the offer zone.

Because path trajectories can reveal decision-making processes^14^, we examined moment-by moment body positions during offer-zone decisions. We found that mice often oriented first toward entering the wait zone before pausing, re-orienting, and then ultimately deciding to skip (Fig. 2a, b). This behavior is a well-studied decision-making phenomenon termed vicarious trial and error (VTE) that reveals on-going deliberation and planning during moments of indecision (Supplementary Discussion)^14–16^. We measured VTE as the absolute integrated angular velocity over the course of a given path trajectory (IdPhi, Supplementary Methods). There was more VTE (IdPhi was larger) during skip decisions in general and particularly so when skipping in more-preferred restaurants (Fig. 2a, d, Supplementary Fig. 4). The presence of VTE suggests that in the offer zone, decisions to skip included a delayed valuation that overrode initial rapid decisions. This provides a potential point of decision-making vulnerability or impairment in self-control—one rooted in failure of a deliberative or planning process when engaged in conflict between a highly desirable reward vs. choosing smarter alternatives—that could be exploited by drugs of abuse.

Interestingly, skipping offers above wait-zone thresholds was more likely to occur the more an animal displayed VTE behavior (Fig. 2e). This suggests that the more a planning process was engaged, the less likely desired rewards could out-compete making smarter choices, independent of offer value (Supplementary Fig. 5). By classifying the amount of VTE required to skip these economic scenarios at least 50% of the time, we found that skipping high delays in more-preferred restaurants required greater amounts of VTE (Fig. 2g). Furthermore, we found enters for offers above versus below wait thresholds were both rapid and indistinguishable in reaction time and VTE (Fig. 2h–k), suggesting reward-taking behaviors were generally snap judgments while reward-opposing behaviors were not.

As noted, mice were more likely to err by entering offers above wait-zone threshold in more- vs. less-preferred restaurants (Fig. 2f). In the wait zone, mice were more likely to quit after enters above than after enters below wait-zone threshold. Moreover, they were more likely to quit while the amount of countdown time left remaining was still above the wait-zone threshold (Fig. 2l, Supplementary Fig. 6). Thus, wait-zone decisions to quit were advantageous change-of-mind re-evaluations correcting economically unfavorable rapid valuations made in the offer zone. This reveals that mice, despite making economically unfavorable decisions in the offer zone, could remediate those initial snap judgments.

We found that mice took longer to quit in more-preferred restaurants (Fig. 2m), indicating changing one’s mind was a tougher decision for highly desired rewards. In fact, mice were less capable of choosing to quit before crossing wait-zone thresholds in more-preferred restaurants (Fig. 2n). This provides a second potential point of decision-making vulnerability in value conflict between desire and choosing smarter alternatives when re-evaluating and changing one’s mind that could also be exploited by drugs of abuse.

### Lasting effects of cocaine or morphine on distinct valuations

Rather than model addiction as maladaptive behaviors in direct pursuit of drug, we used the complex economic behaviors in this task to model the sophisticated level of decision conflict that human addicts often struggle with—the conflict between wanting on the one hand vs. knowing better on the other hand. To test how drugs of abuse can exploit these types of potential decision-making vulnerabilities, well-trained mice after 70 consecutive days of Restaurant Row received either repeated cocaine, morphine, or saline experimenter-administered injections 4 h after each Restaurant Row session that produced psychomotor sensitization (Fig. 1a, Supplementary Fig. 7, Supplementary Methods, Supplementary Discussion)—an escalated locomotor response to repeated drug exposure that has been shown to serve as a behavioral correlate of neural plasticity in cortical and mesolimbic pathways, bio-markers of which in humans are predictive of relapse susceptibility^9,17,18^. Thus, we focused on a timepoint of 2–3 weeks of prolonged abstinence to model the enduring effects of drug use on decision-making processes. Importantly, we did not observe any gross locomotor effects or overall changes in food intake (Supplementary Fig. 8).

**Fig. 3:**
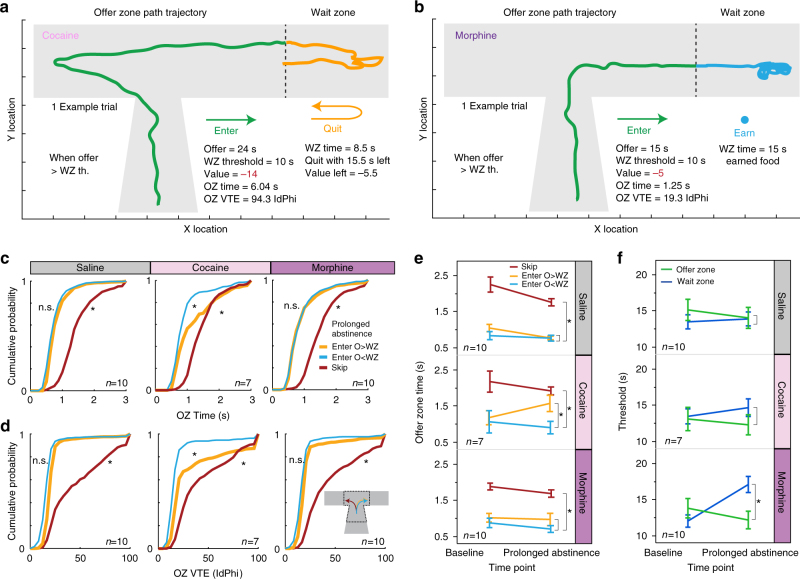
The effects of prolonged abstinence from repeated drug exposure on choice conflict. **a**, **b** Example of path trajectory in the offer and wait zones for negatively valued economically unfavorable offers. **a** A mouse with a history of repeated cocaine exposure initially oriented toward skipping (left) then ultimately re-oriented to enter (right). Capability of quitting was unaltered. **b** A mouse with history of repeated morphine exposure was less capable of quitting rapidly accepted offers. **c**, **d** Cumulative probability distributions of offer-zone time (**c**) and VTE (**d**) for skips as well as enters split by offer value separated by drug-treatment conditions. Both types of enter decisions were rapid compared to skips and indistinguishable from each other for saline and morphine mice (KS tests, not significant, n.s., *P* > 0.05). Cocaine mice displayed increased time and VTE before accepting negatively valued offers (KS tests, *P < 0.05). **e**, **f** Repeated measures Friedman tests correcting for multiple post-hoc Mann–Whitney tests reveal cocaine-specific changes (**e**) in offer zone deliberation time when entering economically disadvantageous offers and morphine-specific changes (**f**) increasing wait-zone thresholds (*P < 0.05). Error bars. ± 1 s.e.m. *N* per group (saline *N* = 10, cocaine *N* = 7, morphine *N* = 10) listed on respective plots

Interestingly, we found that offer-zone time and VTE were disrupted following prolonged abstinence from repeated cocaine but not morphine or saline exposure (Fig. 3a–d). Cocaine-abstinent mice showed increased deliberation behavior before entering offers greater than wait-zone thresholds, inverting the normal behavior (Fig. 3a–e, compare Fig. 2i, Supplementary Fig. 11). Cocaine-abstinent mice initially oriented toward skipping these offers, and then re-oriented to accept them anyway (Fig. 3a). This suggests that cocaine-abstinent mice accepted costly offers despite engaging in VTE and deliberating about turning them down.

In contrast, morphine-abstinent mice had a significant increase in wait-zone thresholds compared to baseline, while cocaine-abstinent and saline-treated mice did not (Fig. 3f). Morphine-abstinent mice also showed increased wait zone thresholds compared to saline-treated mice as well as compared to their own offer zone thresholds (Fig. 3f) This is noteworthy because, while morphine-abstinent mice did not differ in making snap judgments to rapidly accept expensive offers (Fig. 3c–e), they were less likely to correct those economic violations in the wait zone in contrast to the saline and cocaine groups (Fig. 3a, b, f). Thus, probability of quitting significantly decreased (Supplementary Fig. 8A). If morphine-abstinent mice did quit, they took significantly longer to do so (Supplementary Fig. 8B). Neither cocaine- nor morphine-related effects appeared after a single drug exposure and was only apparent following abstinence from repeated drug exposure (Supplementary Fig. 9, Supplementary Discussion). Furthermore, devaluation probe sessions using a flavor-specific pre-feeding procedure revealed flexible decision processes were separately employed in the offer zone and wait zone by all animals but differentially influenced depending on history of cocaine or morphine exposure (Supplementary Fig. 10, Supplementary Discussion).

## Discussion

Recent findings have suggested that choosing between distant options accesses different valuation processes than choosing to opt out from remaining committed to already accepted offers^19^. We can model such decision framings as fundamentally distinct types of intertemporal choice modalities.

**Fig. 4:**
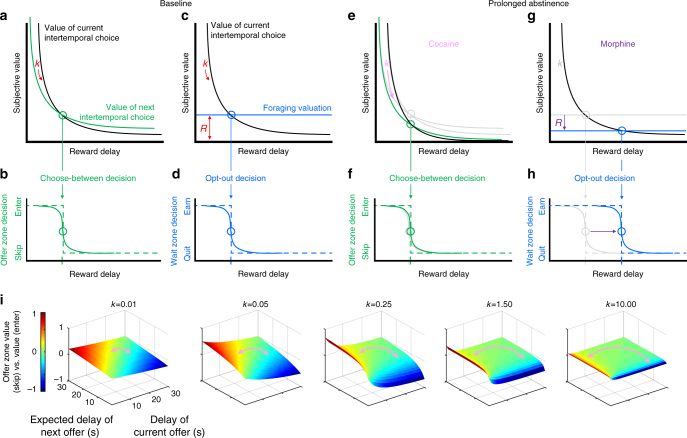
Neuroeconomic modeling of separable computation-specific changes in decision conflict valuation algorithms. **a**–**d** Baseline. **a** Deliberative model: hyperbolic temporal discounting function of the current choice (black) is compared against a second hyperbolic temporally discounted function of the expected next choice (green), with a discounting rate *k* (red). **b** Offer zone choose-between thresholds are derived from this intersection. **c** Foraging model: hyperbolic temporal discounting function (black) of work remaining with discounting rate *k* (red) is compared against the average opportunity cost of reward availability in the rest of the environment, *y*-intercept *R* (red). **d** Wait-zone opt-out thresholds are derived from this intersection. **e**–**i** Modeling the effect of our drug delivery and forced abstinence manipulation. **e** Our data in mice with a history of repeated cocaine exposure are consistent with an increase in the *k* parameter in offer-zone deliberative valuation model, which yields no change in offer-zone thresholds (**f**), but yields increased indecision particularly for economically unfavorable high cost offers (**i**). **g** Our data in mice with a history of repeated morphine exposure are consistent with a decrease in the *R* parameter in the wait-zone foraging valuation model, which leads to an increase in the wait-zone threshold (**h**)

Because VTE behavior occurs in the offer zone, particularly when skipping expensive offers, animals are likely to be engaged in episodic future thinking and deliberation to search and plan for better offers that may lie ahead and resist accepting immediately available highly desired rewards^14^. During VTE, hippocampal representations sweep forward along the path of the animal, alternating between potential goals^20^. Such goal representations are synchronized to reward value representations in the prefrontal cortex and ventral striatum, suggesting outcome predictions are being evaluated serially during VTE^21,22^. This is dissociable from dorsal striatum valuations that occur during rapid decisions when VTE is not engaged^23^. To this end, we modeled two hyperbolic functions discounting the value of the known current and expected next alternative where the discounting rate for an individual is represented by *k*. The decision change occurs at the intersection of these two hyperbolic functions (Fig. 4a). This well-established neuroeconomic model of choosing between alternatives^24–26^ underlies the offer-zone threshold valuation measured on our task (Fig. 4b).

In contrast, quitting the wait zone is an opt-out decision. Such judgments appear in well-studied decision processes common in foraging paradigms^19,27–29^. This can be modeled as a comparison of the hyperbolic temporally discounted value of work remaining compared against the average opportunity cost of reward availability in the rest of the environment (*R*, Fig. 4c). The intersection of this comparison underlies the wait-zone threshold valuation measured on our task (Fig. 4d).

In deliberative models, studies have modeled changes in the hyperbolic discounting rate *k* in drug users as steeper, thus over-valuing immediate rewards^30^. These tasks, however, measure *k* as a product of the outcomes chosen and do not typically characterize the deliberation behaviors that led up to the outcomes selected. Other theories in foraging models have proposed that drug users experience a re-normalization of the average available reward in the world where *R* decreases and thus decreases the value of alternative options in the rest of the environment^8^. Importantly, economic theory suggests that both of these valuation changes (an increase in *k* or a decrease in *R*) could drive recovering addicts to make bad decisions and relapse^2^.

Our data revealed no changes in either the offer-zone or wait-zone threshold in cocaine-abstinent animals. From this, we must conclude that whatever decision-making changes occurred in the cocaine-abstinent animals, it did not shift the crossover points in deliberative or foraging valuation algorithms. What we did find is an increase in offer-zone deliberations for costly offers. This effect could occur as a consequence of a change (increase) in offer-zone choose-between hyperbolic discounting rate *k* (Fig. 4e, f, i). An increase in *k* in both hyperbolic curves in a deliberative model can change the shape of the curves without changing the crossover point. Because hyperbolic discounting curves decrease in steepness as one moves out along the curve, this would effectively decrease discriminatory resolution when choosing between costly offers (Fig. 4i). We argue this is why cocaine-abstinent mice struggled before giving in to accepting expensive offers anyway despite deliberating.

Our data revealed no change in the offer-zone threshold, but did find a right shift in the wait-zone threshold of morphine-abstinent animals. This cannot occur due to an increase in the hyperbolic discounting rate *k* because such a change in a foraging model would shift the crossover point to the left and decrease the wait-zone threshold, which is the opposite of our observed behavioral findings (Fig. 4c, d). Instead, in a foraging model, a decrease in *R* or the average expected value in the rest of the environment relative to a given reward opportunity would shift the crossover point to the right only in the wait zone. Thus, we argue that this right shift in the willingness to wait out a delay once started in the wait zone is due to the effect of morphine diminishing the average rate of reward *R* expected in the world (Fig. 4g, h). This concept is consistent with recent theories of opioid abuse that suggest other rewards in the world are re-normalized and pale in comparison after having experienced morphine^2^. Taken together, we highlight two dissociable points of failure in decision making exploited uniquely by two drugs of abuse—before making bad deliberative judgments versus re-evaluations after making bad snap judgments.

These findings are particularly relevant to a timepoint when recovering addicts who are on the verge of relapse struggle with making the right decisions. Our work highlights the notion that complex valuation processes can be carefully modeled in animal behavior. Disruptions in deliberative processes separate from foraging processes can suggest distinct circuit-specific computations that can go awry in different forms of addiction.

Many studies examining the lasting neurobiological changes induced by different drugs of abuse, including psychostimulants and opioids, generally propose a unified theory of addiction common to most abused substances that converges on overlapping changes in synaptic plasticity within the mesolimbic reward system^31^. The majority of these studies focus on changes in glutamatergic and dopaminergic signaling in the ventral tegmental area and nucleus accumbens^31^. However, there are reports of contrasting or opposing lasting neurobiological changes induced by cocaine and morphine, including differential effects on accumbens spine density, synaptic remodeling, and gene expression^32–35^. We suggest that taking into account the information processed within these circuits as well as other circuits during discrete aspects of decision-making computations is critical in order to understand multi-faceted, potentially dysfunctional valuation processes that can ultimately drive addiction-related behaviors.

Our data uncover unique computation-specific etiologies separated within the same trial that may be underlying different forms of addiction that more traditional behavioral paradigms may not be sensitive enough to detect. We propose that computation-specific therapeutic interventions are likely necessary to ameliorate addiction subtypes that disrupt, in different ways, the decision to use despite knowing better.

## Methods

### Mice and training

32-C57BL/J6 male mice, 13 weeks old, were initially trained in Restaurant Row. Mice were single-housed at 11 weeks of age in a temperature- and humidity-controlled environment with a 12-h-light/12-h-dark cycle with water ad libitum. Mice were food restricted and trained to earn their entire day’s food ration during their 1 h Restaurant Row session. Experiments were approved by the University of Minnesota Institutional Animal Care and Use Committee (IACUC; protocol number 1412A-32172) and adhered to the National Institutes of Health (NIH) guidelines. Mice were tested at the same time every day in a dimly lit room, were weighed before and after every testing session, and were fed a small post-session ration in a separate waiting chamber on rare occasions to prevent extremely low weights according to IACUC standards (not <85% free-feeding weights). Reliable behavioral measures were previously achieved on this task with sample sizes as small as five animals. Therefore, we ensured that sample sizes were no smaller than 7 animals, even after attrition. We started with 32 mice. One mouse died before treatment assignment and is not included in any analysis; three mice were lost due to cocaine and are not included in any cocaine-related comparisons. Analyses across time include the same animals. No data points were removed due to outliers.

### Drug exposure

Animals were randomly assigned to receive either saline, cocaine, or morphine treatments, counterbalancing groups across as many behavioral parameters as possible. After 70 days of training mice were injected with saline (0.9% NaCl) for 3 days in order to get them acclimated to the stress of injections. Restaurant Row testing took place during the day during their light phase. Only on special days when injections were to be administered, these took place in the dark phase in the evening after Restaurant Row testing for that day completed. Acute injection-induced locomotor activity was monitored in the 90 min immediately following drug injections in a separate locomotion chamber, not in the Restaurant Row apparatus. All injections were volume corrected after measuring mouse body weights right before injections. Next, mice received 12 evenings of repeated drug or saline control injections. This is a standard and well-established drug-treatment regimen known to produce robust and long-lasting drug-related changes, particularly after prolonged abstinence, to model a behavioral stage just before relapse. Overall, our goal was to measure how decision processes were affected by repeated drug use, rather than acutely when animals were on drug. Thus, it is the prolonged abstinence timepoint ~2 weeks following the 12th drug injection that is of importance. Experimenters that handled animals during Restaurant Row testing were blinded to drug group. Behavior testing in Restaurant Row was fully automated. Behaviorally analyses were also automated across all animals using Matlab.

### Statistical analyses

All statistical analyses were carried out using JMP Pro 13 Statistical Discovery software package from SAS. Statistical significance was assessed using non-parametric statistical tests, as the data were not normally distributed (offer-zone time, offer-zone VTE, wait-zone quit time, post-earn linger time, and offer- and wait-zone thresholds all reject normal distributions using the Kolmogorov–Smirnov–Lilliefors test for goodness of fit, *P* < 0.01). Described below are the statistics used for each main figure, where applicable. Statistics for Supplementary Figures are detailed in corresponding figure captions or in the Supplementary Discussion. All error bars are expressed as ±1 s.e.m. Asterisks used in figures are intended to direct attention to comparisons of interest.

### Main figure statistics

Figures 1a–c, e, 2a, b, e, 2h, i, 3a, b, and 4a–i are illustrative in nature, single-session examples, or intended to demonstrate derivation of a higher-order metric summarized for comparison in a separate figure, and thus analyses reports are deemed not appropriate or not included.

The Kruskal–Wallis (KW) test was used as a non-parametric equivalent to the parametric one-way analysis of variance (ANOVA) test in Figs. 1d, f, g, 2c, d, f, g, m, n to test dependent measures against flavor rankings (or against the three conditions described in Fig. 2l). Post-hoc analyses controlling for multiple comparisons were performed using Dunn’s test to preserve pooled variance from the KW test in order to compare conditions in a pairwise manner. Much of these comparisons included testing flavor rankings pairwise (e.g., most-preferred to least-preferred) as well as to compare values of the same flavor ranking across levels of an separate factor stated on each figure (e.g., skip vs. enter, offer zone vs. wait zone). KW tests were significant across rank on all metrics in the above figures (*P* < 0.0001) except in Fig. 2c, d for the enter condition (*P* > 0.05). Dunn’s tests showed that the most-preferred flavor was significantly greater than the least-preferred flavor on all metrics in the above figures (*P < 0.0001). Dunn’s test also showed that offer-zone thresholds and slope were greater than wait-zone thresholds and slope (Fig. 1f, g, *P < 0.0001), except between threshold types in least-preferred restaurants (Fig. 1f, *P* > 0.05). Dunn’s test also showed that skips were greater than enters in both offer-zone time and VTE in all restaurants (Fig. 2c, d, *P < 0.0001). Lastly, KW and Dunn’s tests on quitting behavior in Fig. 2l confirm economically efficient quits made up the majority of quit events in the wait zone (*P < 0.0001).

In addition to the significant interactions across rank in Fig. 2f, n, the Sign test was used to assess if behavior in each restaurant was above or below the 1:1 ratio line on economic inefficiency in the offer zone (Fig. 2f) and the wait zone (Fig. 2n). Data above the 1:1 ratio line, or a positive sign, indicate economically inefficient behavior. Only behavior in the offer zone of the most-preferred flavor was above the 1:1 ratio line (Fig. 2f, *P* < 0.0001), and not for other flavors in the offer zone nor any flavor in the wait zone (Fig. 2n, *P* > 0.05).

The Kolmogorov–Smirnov test was used to assess differences in cumulative probability distributions of offer-zone time and VTE in Figs. 2j, kj–k and 3c, d. Our comparison of interest was between enters for offers above wait-zone threshold and enters for offers below wait-zone threshold, which at baseline were not statistically different from each other in both time and VTE (Fig. 2j, k, *P* > 0.05). This was replicated at the prolonged abstinence timepoint in both the saline and morphine groups (*P* *>* 0.05), but not cocaine group (*P < 0.01) for both offer-zone time and VTE (Fig. 3c, d).

The Friedman test was used as a non-parametric equivalent to the parametric one-way ANOVA with repeated measures in Fig. 3e, f when comparing behaviors across two timepoints (baseline and prolonged abstinence). Only in the cocaine group did offer-zone deliberations when entering expensive offers increase. Simulations controlling for differences in offer distributions were run in Supplementary Fig. 11. Only in the morphine group did wait-zone thresholds significantly increase across timepoints (*P < 0.05), while offer-zone thresholds did not, nor either threshold in the saline and cocaine groups (*P* > 0.05). Post-hoc analyses using Mann–Whitney tests while correcting for multiple comparisons allowed for non-parametric comparisons at either timepoint between offer-zone and wait-zone behaviors between decision types or between drug conditions. At the prolonged abstinence timepoint, in the morphine group, wait-zone thresholds were significantly higher than offer-zone thresholds (*P < 0.05), which were no different at baseline or at either timepoint in the saline and cocaine groups (*P* > 0.05). Lastly, wait-zone thresholds at the prolonged abstinence timepoint in the morphine group was significantly higher than the saline group (*P < 0.05), while comparisons of wait-zone thresholds between cocaine and saline animals were no different at the prolonged abstinence timepoint (*P* > 0.05).

### Modeling

The model in Fig. 4i was generated via Matlab simulations where we calculated the probability of entering vs. skipping offers as a function of increasing delays from 1 to 30 s of two offers (the current offer (*d*_1_), and the expected next offer (*d*_2_)). Each panel shows how the shape of the value function (*V* = 1/(1 + *k* × *d*_1_) – 1/(1 + *k* × *d*_2_)) changes with increasing *k* (increasing impulsively hyperbolic functions).

For additional information see Supplementary Methods.

### Data availability

Data available on request from the authors.

## Electronic supplementary material

Supplementary Information
