# Supplementary material for: Prolonged abstinence from cocaine or morphine disrupts separable valuations during decision conflict
Source: Nat Commun. 2018 Jun 28;9:2521. doi: 10.1038/s41467-018-04967-2 (PMC6023899; doi:10.1038/s41467-018-04967-2)
Supplement: Supplementary file 1 — Supplementary Information [file 41467_2018_4967_MOESM1_ESM.pdf]

Supplementary Information

Prolonged abstinence from cocaine or morphine disrupts separable valuations during decision conflict

Sweis et al.

Included in this document:

Supplementary Methods

Supplementary Discussion

Supplementary Figures 1-11

Supplementary References

Supplementary Methods

Pellet training
Mice underwent 1-week of pellet training before being introduced to the Restaurant Row maze. During this period, mice were taken off of regular chow and introduced to a single daily serving of BioServ full nutrition 20mg pellets in excess (5g). This serving consisted of a mixture of chocolate-, banana-, grape-, and plain-flavored pellets. Next, mice (hungry, before being fed their daily ration) were introduced to the Restaurant Row maze 1 day prior to the start of training and were allowed to roam freely for 15min to explore, get comfortable with the maze, and familiarize themselves with the feeding sites. Restaurants were marked with unique spatial cues. Feeding bowls in each restaurant were filled with excess food on this introduction day.

Restaurant Row training
Task training was broken into 4 stages. Each daily session lasted for 1hr. At test start, one restaurant was randomly selected to be the starting restaurant where an offer was made if mice entered that restaurant's T-shaped offer-zone from the appropriate direction in a counter-clockwise manner. During the first stage (day 1-7), mice were trained for 1 week being given only 1s offers. Brief low pitch tones (4000Hz, 500ms) sounded upon entry into the offer-zone and repeated every second until mice skipped or until mice entered the wait-zone after which a pellet was dispensed. To discourage mice from leaving earned pellets uneaten, motorized feeding bowls cleared any uneaten pellets upon wait-zone exit. Left over pellets were counted after each session and mice quickly learned to not leave the reward site without consuming earned pellets. The next restaurant in the counter-clockwise sequence was always and only the next available restaurant where an offer could be made such that mice learned to run laps encountering offers across all four restaurants in a fixed order serially in a single lap. Mice quickly learned not to run in the incorrect direction. During the second stage (day 8-12), mice were given offers that ranged from 1s to 5s (4000Hz to 5548Hz, in 387Hz steps) for 5 days. Offers were pseudo-randomly selected such that all 5 offer lengths were encountered in 5 consecutive trials before being re-shuffled, selected independently between restaurants. Again, offer tones repeated every second in the offer-zone indefinitely until either a skip or enter decision was made. In this stage and subsequent stages, in the wait-zone, 500ms tones descended in pitch every second by 387Hz steps counting down to pellet delivery. If the wait-zone was exited at any point during the countdown, the tone ceased and the trial ended, forcing mice to proceed to the next restaurant. Stage 3 (day 13-17) consisted of offers from 1s to 15s (4000Hz to 9418Hz) for another 5 days. Stage 4 (day 18-70) offers ranged from 1s to 30s (4000Hz to 15223Hz) and lasted until mice showed stable economic behaviors. We used 4 Audiotek tweeters positioned next to each restaurant powered by Lepy amplifiers to play local tones at 70dB in each restaurant. We recorded speaker quality to verify frequency playback fidelity. We used Med Associates 20mg feeder pellet dispensers and 3D-printed feeding bowl receptacles fashioned with mini-servos to control automated clearance of uneaten pellets. Animal tracking, task programming, and maze operation was powered by AnyMaze (Stoelting).

Restaurant Row Metrics
Vicarious trial and error behavior (VTE) was measured as the absolute integrated angular velocity of a mouse's x and y position over the course of time and distance from tone-onset upon entry into the offer-zone until exiting the offer-zone (either toward the wait-zone or toward the

corridor heading to the next restaurant). From this, we could capture the degree to which animals interrupted smooth offer zone passes with pause-and-look re-orientation behaviors, known as vicarious trial and error (VTE).[1] The physical hemming-and-hawing characteristic of VTE is best measured by calculating changes in velocity vectors of discrete body x and *y* positions over time as *dx* and *dy*. From this, we can calculate the momentary change in angle, *Phi*, as *dPhi*. When this metric is integrated over the duration of the pass through the offer zone, VTE is measured in the offer zone as the absolute integrated angular velocity, or *IdPhi*, until either a skip or enter decision was made. Reaction time in the offer-zone was also measured in this period.

Reaction time to quit was also measured in the wait-zone from tone-count-down-onset until exit from the wait-zone prematurely before a pellet is earned. Post-earn-consumption-and-lingering-time was measured from pellet delivery-onset until the first exit was made out of wait-zone. In an earlier pilot study, cameras were placed in the wait-zone in order to observe lingering behaviors. After immediate pellet consumption, mice exhibited no unusual behaviors other than occasional grooming and checking the empty pellet receptacle for varying lengths of time before exiting and proceeding to the next restaurant.

Offer- and wait-zone thresholds were measured for each session by fitting sigmoid functions to zone choice outcomes as a function of offer delay, restaurant by restaurant. Inflection point and slope of each sigmoid fit was calculated. In order to calculate the value of the offer on any given trial, thresholds were re-calculated in a leave-one-out analysis excluding the current trial. We then used wait-zone threshold minus offer to calculate value.

Economic conflict inefficiency (Fig. 2F,2N) was measured both for the offer-zone (Fig. 2F) and wait-zone (Fig. 2N). This metric characterized how mice responded to an economically unfavorable offer (an offer where the delay was greater than wait-zone threshold). The ratio of the probability of entering the wait-zone for offers above the wait-zone threshold relative to skipping them was calculated in each restaurant as a function of rank. Similarly, in the wait-zone, after mice had already accepted such offers greater than wait-zone threshold, we characterized how long it took an animal to quit such an offer. If mice took so long that the amount of time remaining when quitting was less than wait-zone threshold, that was characterized as an economically inefficient quit. The ratio of the probability of quitting these offers after they counted down passed wait-zone thresholds relative to quitting before the countdown passed wait-zone thresholds was calculated in each restaurant as a function of rank.

In order to control for the possibility that the analysis of changes in VTE in the offer-zone in economically unfavorable acceptances (taking offer-zone deals that are above the wait-zone thresholds) could have been affected by unequal or different distributions of offers based on trial type (e.g., skipping offers, entering offers above threshold, or entering offers below threshold), we generated simulated shuffled data sets of reaction time and VTE when both skipping and entering offers below threshold matching the same trial-by-trial distributions of offer lengths as those subsets of trials where mice entered offers above threshold. In Fig. 2J-K and Fig. 3C-D, this ensures any changes seen in offer-zone behaviors, particularly when entering economically favorable vs. unfavorable offers, are not skewed by differences in distribution of trials of different offer lengths (Supplementary Fig. 11, Supplemental Discussion).

Drug exposure regimen and locomotor sensitization

This drug treatment regimen is a simple, straightforward yet powerful means of producing robust and long-lasting behavioral and neurobiological changes linked to aspects of addiction such as incentive sensitization and neural plasticity in the mesocorticolimbic dopamine system.[2-4] By looking at a time point during prolonged abstinence, we intended to characterize changes that may reflect the life-long decision-making problems seen in recovering addicts. Long-lasting forms of neurobiological plasticity changes are observed at these prolonged abstinence time points coinciding with and causally linked to escalation of craving. Such plasticity measurements predict relapse susceptibility in human addicts.[5]

Injections took place in the evening 4 hours post-Restaurant Row testing. Our goal was to expose animals to drugs of abuse outside of testing hours, to be especially sure drug has cleared the animals' system before the next day's behavior. Furthermore, we wanted to avoid the effects of acute withdrawal on each day of Restaurant Row testing during the drug exposure phase. Repeated Restaurant Row testing during the drug exposure phase was not intended to capture instances when drug is on board, nor was it intended to compare changes between first and subsequent drug exposures, nor was it intended to analyze the effects of immediate cessation of repeated drug administration on decision-making. Instead, the goal was to interrogate decision-making after prolonged abstinence. Repeated Restaurant Row testing during the drug exposure phase and early abstinence was mainly intended to (1) ensure the animals did not unlearn the task day to day, and (2) maintain regular self-earned food-intake amounts contingent upon task performance rather than giving the animals non-contingent food or days off.

In the evening at the time of each drug injection, mice were placed in large locomotion monitoring boxes with tracking cameras fixed above automatically measuring distance traveled using AnyMaze software (Stoelting). Mice were placed in the boxes for 20min before being injected intraperitoneally with saline and then monitored for 90min post-injections. Then mice were divided into three groups: saline (n=10), cocaine (n=10), and morphine (n=10). One mouse out of the original 31 was excluded because it never learned the task. Mice were then injected with their respective treatment for 12 consecutive nights while being tested in Restaurant Row regularly. For the drug groups, mice were given lower doses (15mg/kg cocaine, 10mg/kg morphine) on the first and last nights and received repeated higher doses (30mg/kg cocaine, 20mg/kg morphine) on the intermediate 10 nights. Three mice were lost during the drug phase in the cocaine group and were excluded from analyses. Mice were then put through a forced abstinence period for 2 weeks while regularly being tested in Restaurant Row.

In addition to the prolonged abstinence timepoint that is the main focus of the drug paradigm, we also introduced animals to an acute drug challenge at the end of the ~2 weeks of abstinence timepoint. This was intended to probe responsivity to a drug prime and assess degree of locomotor sensitization that typically incubates over prolonged abstinence and can be expressed upon drug-re-exposure. Locomotor sensitization was measured as the psychomotor response measured immediately following drug injection at this timepoint compared to psychomotor response measured immediately following drug injection on the 12th evening of the repeated drug exposure sequence. We randomly injected mice 3 times with saline across the evenings before

experiencing this acute drug challenge, again, to acclimate the animals to the stress of injections in preparation for the forthcoming drug-re-exposure challenge.

Mice were challenged in the evening with a single low dose of drug same dose as the 1st and 12th night of drug in the repeated drug exposure sequence, being re-exposed to the same drug administered previously. Saline mice were divided into two groups of n=5 to receive a low dose of either cocaine or morphine for the first time, acutely. Despite the small sample size, this split was done to ensure that sensitized locomotion in response to a single dose was present only in animals with a history of repeated drug exposure. This comparison was statistically significant even with samples of n=5. This replicates work from our lab and numerous others.[2-4] Regardless, the primary analyses (comparing baseline to prolonged abstinence) occurred before the saline group was split and statistics were done with the complete saline group as control.

Following the acute drug-re-exposure challenge, Restaurant Row was tested regularly during the day for an additional 2-3 weeks. Because there were no lasting drug effects on any animal behavior in the formerly saline animals after the acute drug-re-exposure challenge session which took place ~20 days before the pre-feeding probe sessions (described below), this group served as control conditions for the pre-feeding probe sessions.

Devaluation/Invigoration Pre-feeding Probe Sessions

The pre-feeding probe sessions were performed at the end of the experiment and were intended to elucidate if rapid decisions or snap-judgments were flexible or inflexible processes. Devaluation probes are often used to differentiate goal-oriented (flexible and thus sensitive to devaluation) and habitual (inflexible and thus insensitive to devaluation) decision processes.[6-10] The devaluation probe in our task allowed us to rule-out habitual processes. There was no further testing after the pre-feeding probes as the experiment ended and all mice were retired.

Mice were pre-fed 30-60min before testing in an amount equivalent to what they typically earned in their most-preferred restaurant. Since each animal showed individual revealed preferences (i.e. different animals like different flavors best), we fed each animal its most-preferred flavor on one day and its least-preferred on the next. Since some animals received their most-preferred flavor on the first-day of pre-feeding while others received their least-preferred flavor on the first-day of pre-feeding (randomly selected and counter-balanced), day two of pre-feeding flipped this assignment. There were no order effects and no lasting body weight changes on day one versus day two of pre-feeding, so we pooled together the first and second day of pre-feeding to look at group differences between being fed one's most-preferred flavor versus least-preferred flavor.

The fact that all groups still showed sensitivity to the pre-feeding probe (although with intricate fine-grained differences between groups described in the Supplementary Discussion), we determined that the decision-processes in Restaurant Row remained flexible and had not transitioned to habit-like processes.

Supplementary Discussion

Vicarious trial and error
A key to interpreting parallel competing valuations in our task during decision-conflict between forward-looking planning and immediate desire-driven responding is the presence or absence of a critical behavioral metric – vicarious trial and error (VTE) – which has extensively been studied in a series of proof of principle publications.[1] We know that VTE is a sign of deliberation but VTE has not yet been measured in an addiction model.

In 2007, Johnson and Redish discovered that during VTE, hippocampal representations swept forward along the path of the animal, alternating between potential goals.[11] This key result has been replicated several times. We know that these sequences align to hippocampal theta cycles.[12] That is, they are theta sequences. However, the sequences during VTE sweep farther than during normal navigation.[13] The sequences proceed all the way to the goal.[12] If an animal is going to run past one goal to another one, the sequences run farther to the second goal.[14] They reflect indecision in the animal. An animal that knows where to go does not show VTE and the sequences only sweep forward to the goal the animal is actually going to go to.[11,15-16]

Furthermore, neurophysiologically, during VTE, reward-related representations appear in the nucleus accumbens (ventral striatum)[17-18] and in the orbitofrontal cortex.[19] Both of these results have been replicated.[20] These data suggest that there is an evaluation going along with the prediction in hippocampus. Neurophysiologically, we know that there is a triple dissociation between hippocampus (sweeps during VTE), ventral striatum (reward representations during VTE), and dorsal striatum (no extra activity during VTE, but slowly learned situation-action pairs).[21] As animals develop regular paths and VTE goes away, the dorsal striatum develops task-bracketing wherein activity appears at the start of the ballistic journey.[22] This result has been replicated.[23] In both of these papers, VTE is negatively correlated to the striatal task-bracketing.

Behaviorally, VTE occurs during times when the animal knows the structure of the world, but does not know what to do on it. VTE occurs when the animal is indecisive about goals and when contingencies change.[19,23-25] Manipulations that force flexibility in tasks lead to an increase in VTE, while manipulations that force regularity in paths lead to a decrease in VTE.[26] Finally, on tasks able to differentiate decisions that require planning (sometimes called model-based) from decisions that reflect cached values (sometimes called model-free), VTE occurs when the decisions show planning (model-based) and disappear when the decisions reflect cached values (model-free).[24,26-27]

In this task, we can take VTE as a sign of indecision and deliberation, and a lack of VTE as a sign of quick, decisive decisions (snap-judgments). In this task, we can reliably detect the difference between VTE and rapid (snap) judgments. Furthermore, we found that when VTE events took place, they did so with delayed onset overriding initial snap judgments in the offer-zone that would have otherwise violated normative economic behavior. This form of delayed deliberative VTE-containing override decisions rescued and prevented economic violations from occurring, importantly only when skipping, and could serve as a behavioral operationalization of knowing better or should not judgments. Sometimes when such slower deliberative VTE process

failed to come online, mice accepted expensive offers only to later reverse that initial rapid commitment by quitting in the wait-zone. This indicated that a re-evaluation process can also occur in the wait-zone. Both override-processes in the offer-zone or wait-zone took longer to override in higher preferred restaurants, capturing an increasingly stronger desire-component of these parallel computational processes.

Sub-optimality

Theories of foraging behavior are rooted in hypotheses of optimizing time allocation in order to maximize reward rate.[28] In Restaurant Row, all flavored pellets are of equal caloric value, and thus any differences in reinforcement rate as a function of cost between flavors must be taken as reflecting an underlying subjective valuation. Mice demonstrated a large variability in subjective flavor preferences from which we found interesting asymmetries and interactions with multiple valuation processes measurable on this task.

If we take into account individual differences in subjective preferences of willingness to wait for rewards (wait-zone thresholds), we can still determine a measure of sub-optimality, normalized to each animal's idiosyncratic preference for each flavor. In order to calculate maximum number of rewards a mouse could earn in each restaurant taking into account subjective flavor preferences, we simulated Restaurant Row sessions yet eliminated wasteful behaviors. To this end, in this model, we forced offer-zone thresholds to match wait-zone thresholds, thus eliminating all quit events. Furthermore, we eliminated differences in offer-zone deliberation time and post-earn lingering time between flavors (by using minimum deliberation time and minimum consumption time collapsed across all restaurants based on each animal's performance). We also used minimum transit time between restaurants based on each animal. These are the times the animal could have used if the only difference between decisions was the underlying willingness-to-wait thresholds between the flavors.

We found that mice overall were sub-optimal on this metric, even after taking into account individual differences in subjective flavor preferences and that prolonged abstinence from repeated drug exposure did not influence this metric (Supplementary Fig. 8D).

We also found that degree of sub-optimality interacted with flavor ranking. That is, mice were more sub-optimal in less-preferred restaurants. This is likely due to the disproportionate excess amount of time spent in the offer-zone, wait-zone, and lingering in more-preferred restaurants. Such disproportionate excess amount time that was removed from our optimal-performance model, when re-allocated optimally, would lead the model to predict disproportionately higher earnings than actual in less-preferred restaurants. This is due to the combination of excess time available, lower thresholds in those restaurants, and greater likelihood of our model encountering low cost offers in those restaurants that can be earned and that would have not been actually encountered otherwise. Thus, this yielded higher predicted than actual reinforcement rates in less-preferred restaurants.

Drug-related effects

Importantly, our decision-making tests are made during times when cocaine and morphine are not on board, and we show that drug exposure after the drug has cleared the animal's system does

not have any persistent effects on locomotor activity or appetite that could confound our interpretations of our decision-making tests (Supplementary Fig. 7).

Acute locomotor and appetite changes are typical effects when drug is on board and could confound behavioral performance on many tasks. The half-life of cocaine is ~1hr and morphine is ~2hr.[29] We tested mice on our task 10 hours after each drug injection (which took place 4 hours post-testing on our task) and well into prolonged abstinence for 2 weeks where we observed our decision-making conflict changes.

We used the following metrics to test for off-target effects of chronic drug: speed of locomotion on the task, number of completed laps, total amount of food earned and total weight gained. We found no differences in any of these metrics between controls and drug-treated mice (or within individuals) across the entire experiment. This lack of change rules out off-target effects on locomotion or appetite as possible confounding factors for our observed changes in decision-making metrics, including VTE (Supplementary Fig. 7).

Furthermore, our effects of drug on decision-making persist 2 weeks after chronic drug exposure at a time point when long-lasting circuit changes in decision-making-related brain areas including the prefrontal cortex, nucleus accumbens, and hippocampus are known to develop and when psychomotor sensitization is expressed - a hallmark and behavioral correlate of repeated drug-induced incubation of plasticity changes replicated numerous times.[2-4,30-35]

Our repeated drug exposure regimen did induce psychomotor sensitization measured in the 90-minute window following drug administration expressed after prolonged abstinence during a drug challenge (Supplementary Fig. 7).

Long-lasting changes in decision-making conflict were observed only after repeated drug exposure, not after acute one-time drug exposure (Supplementary Fig. 9). We examined behavior during the drug-exposure phase (Fig 1A, **cyan timepoint 1**), during early abstinence (Fig 1A, **cyan timepoint 2**), and following the acute drug-re-exposure change after prolonged abstinence (Fig 1A, **cyan timepoint 3**). The main timepoint of interest was after prolonged abstinence from repeated drug use, a timepoint at which psychomotor sensitization is typically expressed, at which neural plasticity in defined circuits develop, and at which recovering addicts struggle to make good decisions before relapsing.[2-4,30-35] Psychomotor sensitization seen after repeated drug exposure has been shown to be a behavioral correlate of drug-induced neural plasticity in specific mesolimbic and striatal circuits. That is, animals that show heightened locomotor responses to drug injections following repeated administration and incubated over prolonged abstinence show drug-induced circuit plasticity while animals that do not show heightened locomotor responses do not exhibit neural plasticity.[36]

Nonetheless, we present additional data during the drug exposure phase, early abstinence, and following the drug-re-exposure challenge primarily intended to express degree of psychomotor sensitization incubated throughout prolonged abstinence (Supplementary Fig. 9). We found no decision-making changes during Restaurant Row during the drug-exposure phase in offer-zone deliberation behaviors (Supplementary Fig. 9A-B, enters comparison, non-significant, Kolmogorov-Smirnov tests, P>0.05), nor between the first and last (12th) injection during the

drug exposure phase in thresholds (Supplementary Fig. 9C, wait-zone across time, non-significant, Friedman, $P>0.05$), nor in post-earn lingering time (Supplementary Fig. 9D, lingering time across time, non-significant, Friedman, $P>0.05$).

Looking at the early abstinence time point, we found no changes in offer-zone deliberation behaviors (Supplementary Fig. 9E-F, enters comparison, non-significant, Kolmogorov-Smirnov tests, $P>0.05$), nor between baseline and early abstinence in thresholds (Supplementary Fig. 9G, wait-zone across time, non-significant, Friedman, $P>0.05$), nor in post-earn lingering time (Supplementary Fig. 9H, lingering time across time, non-significant, Friedman, $P>0.05$).

Looking immediately following the drug-re-exposure challenge after prolonged abstinence, we only saw the persisting difference in the cocaine group (Supplementary Fig. 9I-J, enters comparison, cocaine group only, significant, Kolmogorov-Smirnov tests, *$P<0.05$, see Fig. 3C-D for comparison). Interestingly, only in mice with a history of repeated drug exposure, and not in formerly saline-treated mice experiencing drug for the first time at the time of the drug challenge, we saw an increase in wait-zone thresholds immediately before and after the drug-re-exposure challenge (Supplementary Fig. 9K, wait-zone across time, cocaine and morphine, Friedman, *$P<0.05$). Interestingly, in all mice following the drug challenge, we found an increase in post-earn lingering time (Supplementary Fig. 9L, lingering time across time, all mice, Friedman, *$P<0.05$).

Taken together, this suggests that the decision-making changes reported in the main text seen in mice with a history of repeated cocaine and morphine exposure were apparent only after prolonged abstinence and not after a single drug-exposure. Interestingly, all mice appeared to increase lingering time regardless of history of drug use following an acute exposure to drug (Supplementary Fig. 9L). This suggests that hedonic valuations of non-drug rewards can be enhanced during acute withdrawal from drug. An acute drug-re-exposure challenge has been shown in the literature to precipitate reinstatement of drug-seeking behavior as a model of provoking relapse as well as induce neural plasticity changes unique from prolonged-abstinence-induced plasticity.[2-4,30-35] While the main focus of this manuscript was not to actually induce relapse, but rather model decision-making changes just before relapse after prolonged abstinence, it is interesting that drug-re-exposure after prolonged abstinence caused changes in wait-zone thresholds only in mice with a history of repeated drug exposure and not in first-time users (saline-pre-treated mice). This sets the stage for further investigation in future studies to more closely examine decision-making changes at secondary timepoint after relapse.

Devaluation

Referring to **cyan timepoint 4** in Fig. 1A and Supplementary Fig. 10, pre-feeding has been shown to change reward seeking behaviors depending on factors including amount pre-fed, instrumental action being assessed, and reward-selective versus reward-nonselective modulation.[6-10] Pre-feeding-induced devaluation of reward-seeking behaviors has been widely used as a way to probe if behaviors are inflexible, stimulus-response-driven, and thus habit-like versus flexible, response-outcome-driven, and thus goal-directed.[6-10] These two potential responses to a devaluation manipulation such as pre-feeding have been shown to separate behaviors that are differentially driven by separable neural circuits.

We pre-fed mice either their least- or most-preferred flavors in an amount that did not disrupt typical number of laps run or pellets earned (Supplementary Fig. 10A-C, C: Friedman, non-significant, $P>0.05$). Bodyweight did significantly increase following pre-feeding but before testing, yet was normalized by the next day (Supplementary Fig. 10D, before and after feedings, Friedman, significant, *$P<0.05$, before feeding across days, Friedman, non-significant, $P>0.05$).

Wait-zone thresholds were devalued (decreased) in saline and cocaine mice while the thresholds of morphine mice did not change (Supplementary Fig. 10F, Sign test, *$P<0.05$). Only when pre-fed their most-preferred flavor were saline mice devalued in the offer-zone as well (Supplementary Fig. 10E, Sign test, *$P<0.05$). Offer-zone thresholds of cocaine mice interestingly increased, suggesting pre-feeding for these animals carried an invigorating-like food-prime component on this aspect of behavior (Supplementary Fig. 10E, Sign test, *$P<0.05$).

In the offer-zone, deliberation time and VTE when skipping or accepting offers below threshold (economically favorable) was unaltered; however, saline mice accepted offers above threshold (economically unfavorable) more slowly when pre-fed their most-preferred flavor (Supplementary Fig. 10G-H, Sign test, *$P<0.05$), suggesting a shift in the balance of valuation functions. Entering offers above threshold however, just as before, took place after little VTE with no further pre-feeding-induced changes, indicating these events were still snap-judgments (did not involve deliberating about correct alternatives, Supplementary Fig. 10H, Sign test, $P>0.05$). Morphine mice responded just as saline mice did while cocaine mice displayed no changes on this metric (Supplementary Fig. 10G-H, Sign test, *$P<0.05$).

Finally, although lingering remained unchanged in saline-treated mice, morphine-abstinent mice showed invigorated (increased) lingering while cocaine-abstinent mice showed the opposite (Supplementary Fig. 10J, Sign test, *$P<0.05$). Additionally, cocaine-abstinent mice displayed less time spent waiting before quitting (Supplementary Fig. 10I, Sign test, *$P<0.05$). Taken together, pre-feeding revealed changes in dissociable valuation algorithms that were blunted or enhanced based on drug history.

Devaluation experiments can modify the incentive value of instrumental actions and reveal specific encoding of emotional states or craving underlying goal-oriented behavior.[28-31] In appetitive tasks, pre-feeding is one way to accomplish this. Taking advantage of the subjective value properties of rewards and different zones, we found that pre-feeding decreased wait-zone thresholds (indicating devaluation) consistent with satiety effects on incentive processes.[29] These effects were not-flavor specific and seemed to affect appetitive reward taking valuation processes in general. However, only when pre-feeding most-preferred flavors did offer-zone thresholds also decrease. This highlights not only a flavor-specific satiety effect consistent with past reports[6-10] but also a subjective value-specific capacity to modify motivational states unique to choose-between decisions involving highly wanted rewards. Pre-feeding seemed to induce invigoration-like effects in drug-treated mice absent in saline-treated mice. In morphine-abstinent mice, we found increased conditioned-place-preference (CPP)-like lingering, which may reflect enhanced craving and explain why their wait-zone thresholds, which were generally insensitive to change, paradoxically opposed satiety-induced devaluation. In contrast, cocaine-abstinent mice, while sensitive to wait-zone threshold devaluation, paradoxically displayed increased offer-zone thresholds. That is, cocaine-abstinent mice were food-primed to over-value

offers in the offer-zone that were exaggeratedly under-valued in the wait-zone. Thus, the hypothesis that cocaine-abstinent mice may be transitioning into a lower value state once in the wait-zone may explain why they were more likely to quit, quit faster, and spend less time lingering, suggesting the predicted value of accepted rewards were less than expected.

Pre-feeding was not intended to assess drug effects but rather to assess decision flexibility and rule out habitual processes. Because there were no lasting drug effects on any behavior in the formerly saline animals after the acute drug-re-exposure challenge session which took place 20 days before the pre-feeding probe sessions, this group served as control conditions for the pre-feeding probe. Again, the fact that all groups still showed sensitivity to the pre-feeding probe (although with intricate fine-grained differences between groups), we determined that the decision-processes in Restaurant Row remained flexible and had not transitioned to habit-like processes.

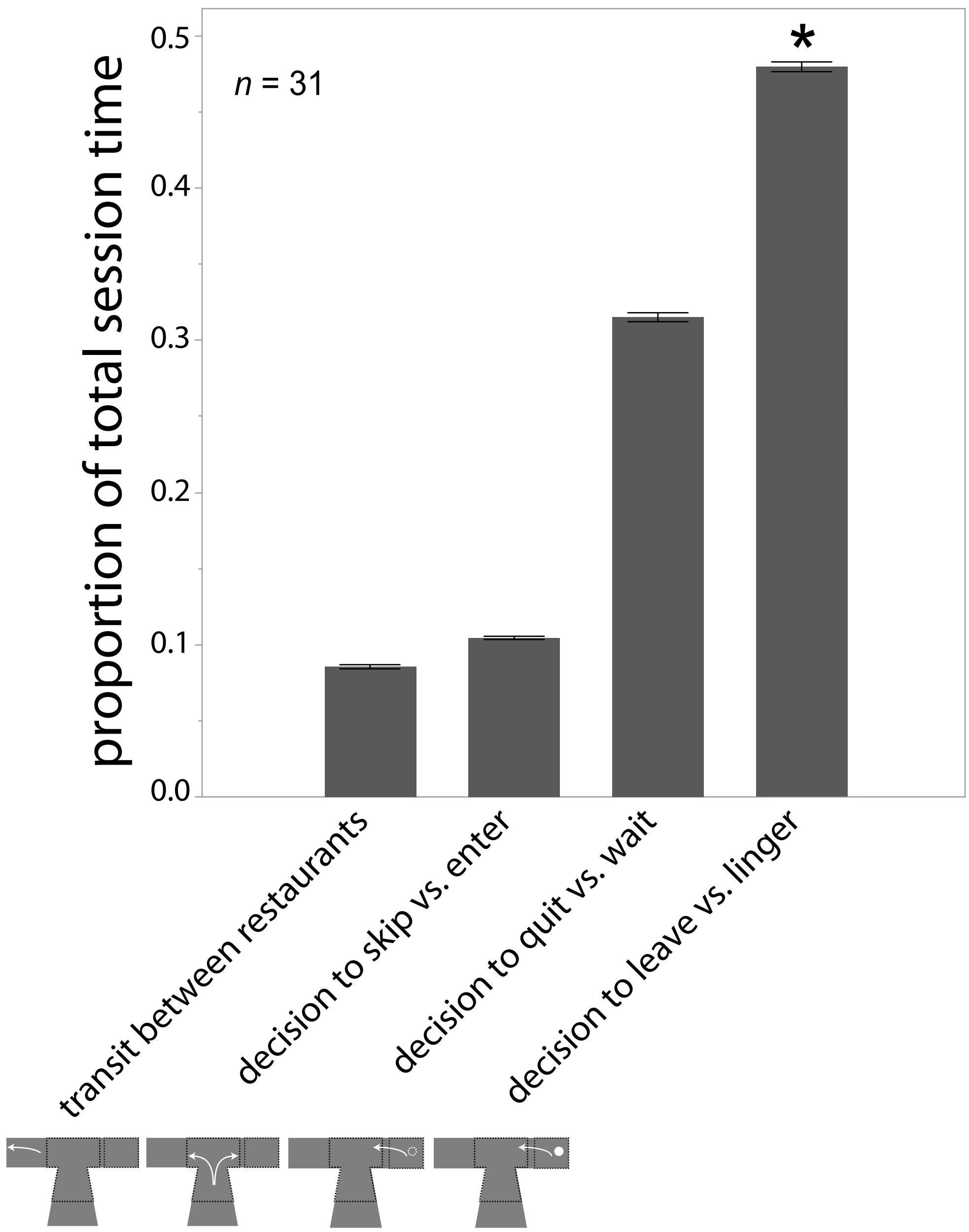

Supplementary Figure 1. Allocation of total session time budget across multiple separate valuation behaviors. (Left to right) Average percent of total session time spent traveling between restaurants, deliberating in the offer-zone (skipping vs. entering, measured between initial tone onset and offer-zone exit), foraging in the wait-zone (investing time before quitting vs. earning pellets, measured between tone countdown onset and premature wait-zone exit or pellet delivery), and consuming food and lingering at the reward-site after earning pellets (measured from time of pellet delivery to wait-zone exit). Majority of total session time was spent lingering at the reward site compared to other task behaviors (Friedman, $P<0.0001$, post-hoc Mann-Whitney comparisons against lingering time, *$P<0.0001$). Error bars. ± 1 SEM. N=31.

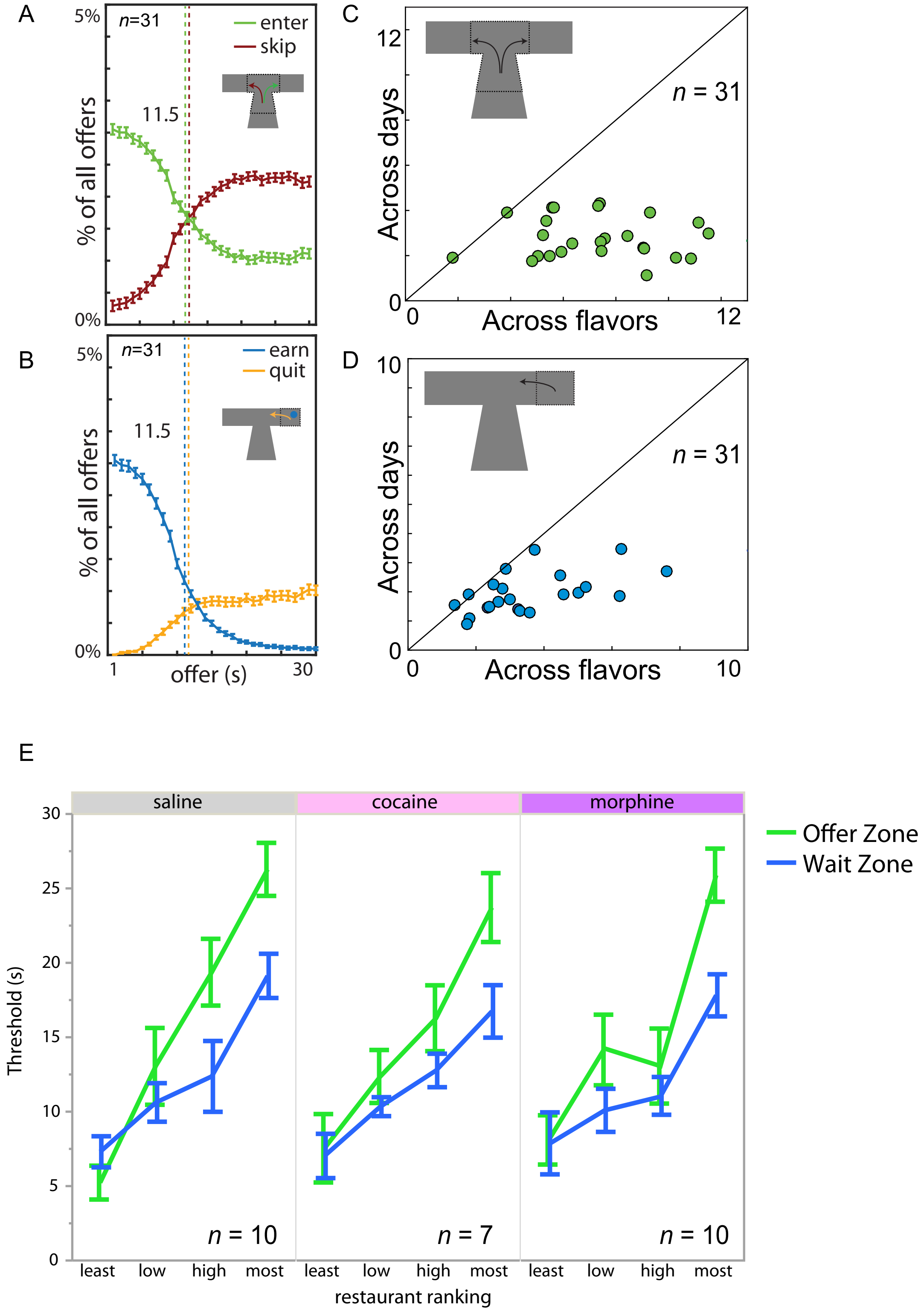

Supplementary Figure 2. Offer discrimination and threshold stability. (A) In the offer-zone, mice accepted (entered) short offers while skipping long offers. (B) In the wait-zone, mice waited for (earned) short offers while quitting long offers. (A-B) Vertical dashed-lines indicates overall threshold collapsed across restaurants (~11.5s in both zones). (C-D) Variability of offer-zone thresholds (C) and wait-zone thresholds (D) was calculated between flavors (x-axis) as well as for a given flavor across 10 days of stable performance (y-axis). Dots represent individual subjects. Space below unity line reflects range of idiosyncratic variability in individual differences in subjective flavor preferences while also reflecting stable preferences within flavor (low relative variability). (E) At baseline, mice assigned to receive either saline, cocaine, or morphine treatments later in the experiment displayed similar trends in offer zone and wait zone thresholds across restaurant rankings. Error bars. ± 1 SEM. Sample size indicated on each plot.

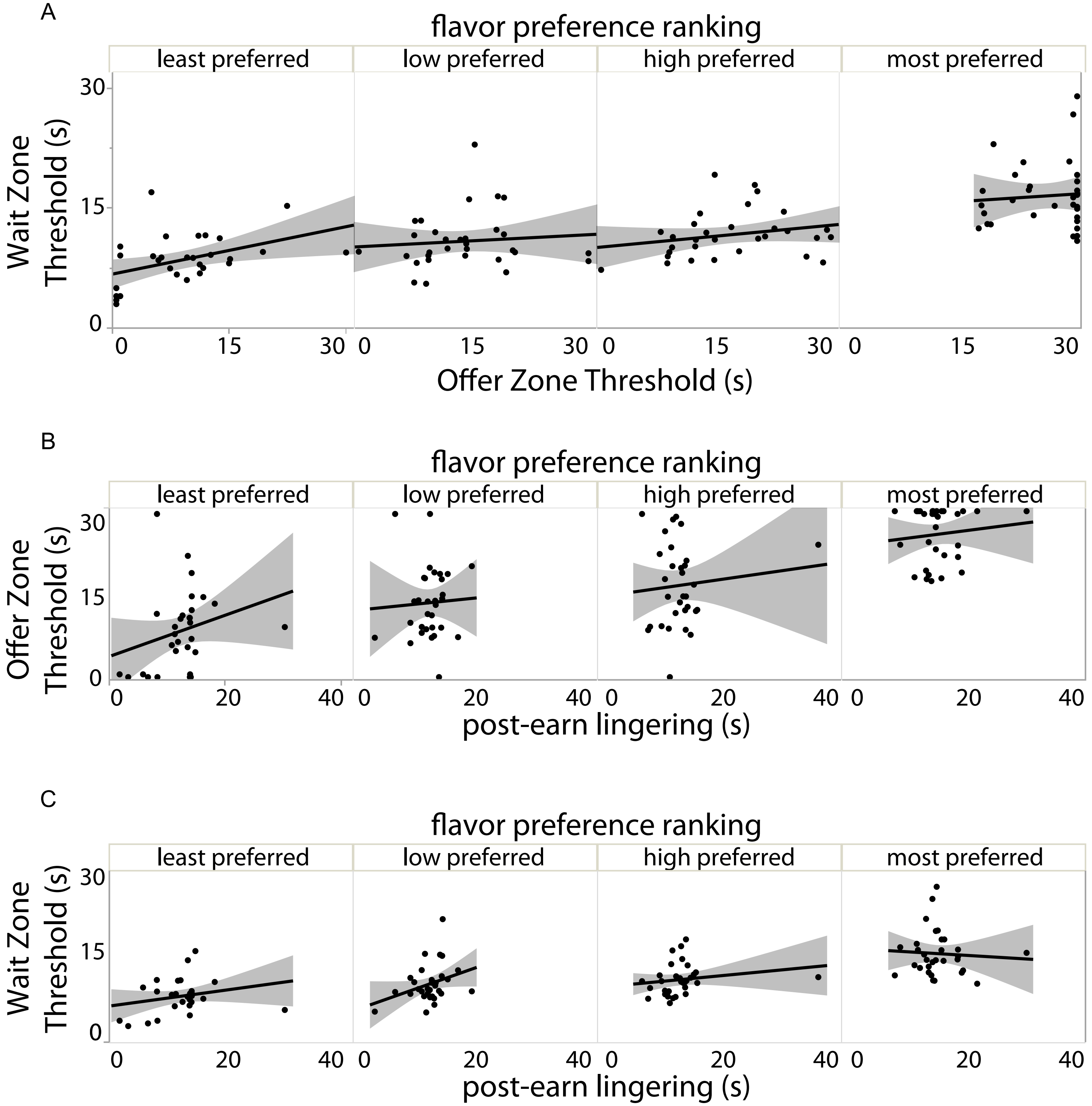

Supplementary Figure 3. Independent and separable valuation metrics across offer-zone, wait-zone, and post-earn lingering behaviors. (A-C) Outside of the ordinal rankings of subjective flavor preferences, no relationships were observed between offer-zone and wait-zone thresholds (A), offer-zone thresholds and post-earn lingering time (B), or wait-zone thresholds and post-earn lingering time. All correlations, correcting for multiple comparisons, resulted in non-significance, P>0.05. (See Fig.1D,1F for agreement that the most-preferred restaurants yielded the highest offer-zone thresholds, wait-zone thresholds, and lingering time). Shaded error region displays 95% CI. N=31.

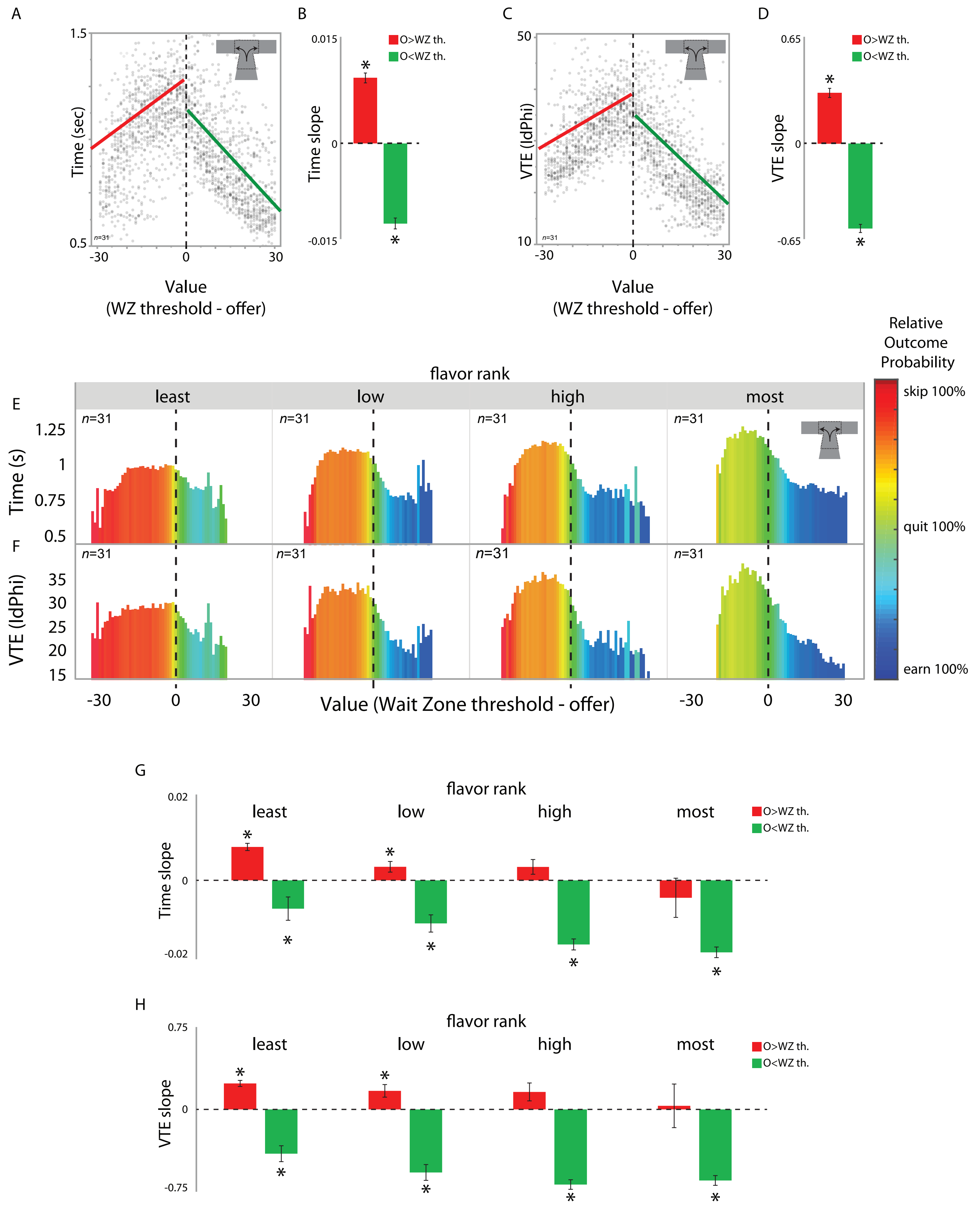
A
1.5
Time (sec)
0.5
n=31
-30
0
30
Value
(WZ threshold - offer)
B
0.015
O>WZ th.
O<WZ th.
Time slope
0
-0.015
*
*
C
50
VTE (IdPhi)
10
n=31
-30
0
30
Value
(WZ threshold - offer)
D
0.65
O>WZ th.
O<WZ th.
VTE slope
0
-0.65
*
*
Relative
Outcome
Probability
skip 100%
quit 100%
earn 100%
flavor rank
least
low
high
most
E
n=31
1.25
1
0.75
0.5
Time (s)
F
35
30
25
20
15
VTE (IdPhi)
-30
0
30
Value (Wait Zone threshold - offer)
G
flavor rank
0.02
0
-0.02
Time slope
least
low
high
most
O>WZ th.
O<WZ th.
H
flavor rank
0.75
0
-0.75
VTE slope
least
low
high
most
O>WZ th.
O<WZ th.

Supplementary Figure 4. Offer-zone deliberation behaviors distributions by value, rank, and trial outcome. (A-D) Linear fits for offer-zone time (A-B) and VTE (C-D) as a function of wait-zone-threshold-derived value revealed decisions got progressively easier (less time and less VTE) when offers were farther away from threshold in either direction (B,D, Sign test slope is significantly different from zero, *$P<0.05$). That is, offers near threshold (zero value, vertical dashed black line) were toughest. (E-H) Same as A-D split by subjective flavor preference rankings. (E-F) Color scale describes the relative likelihood a trial at a given wait-zone-derived value is to end as either a skip, quit, or earn outcome. Note the increasingly sharper leftward color transition toward red (reflecting skip events) for negatively valued offers in less-preferred restaurants compared to the broader leftward color transition that is predominately green for negatively valued offers in more-preferred restaurants (reflecting enter-then-quit events). (G-H) Slopes for both time (G) and VTE (H) for positively valued offers are significantly different (less) than zero in all ranks, while slopes for negatively valued offers only in less-preferred restaurants are significantly different (greater) than zero. This indicates that decisions for worse deals in more-preferred restaurants, unlike in less-preferred restaurants, were not any easier to make. (*$P<0.05$). Error bars. ± 1 SEM. N=31.

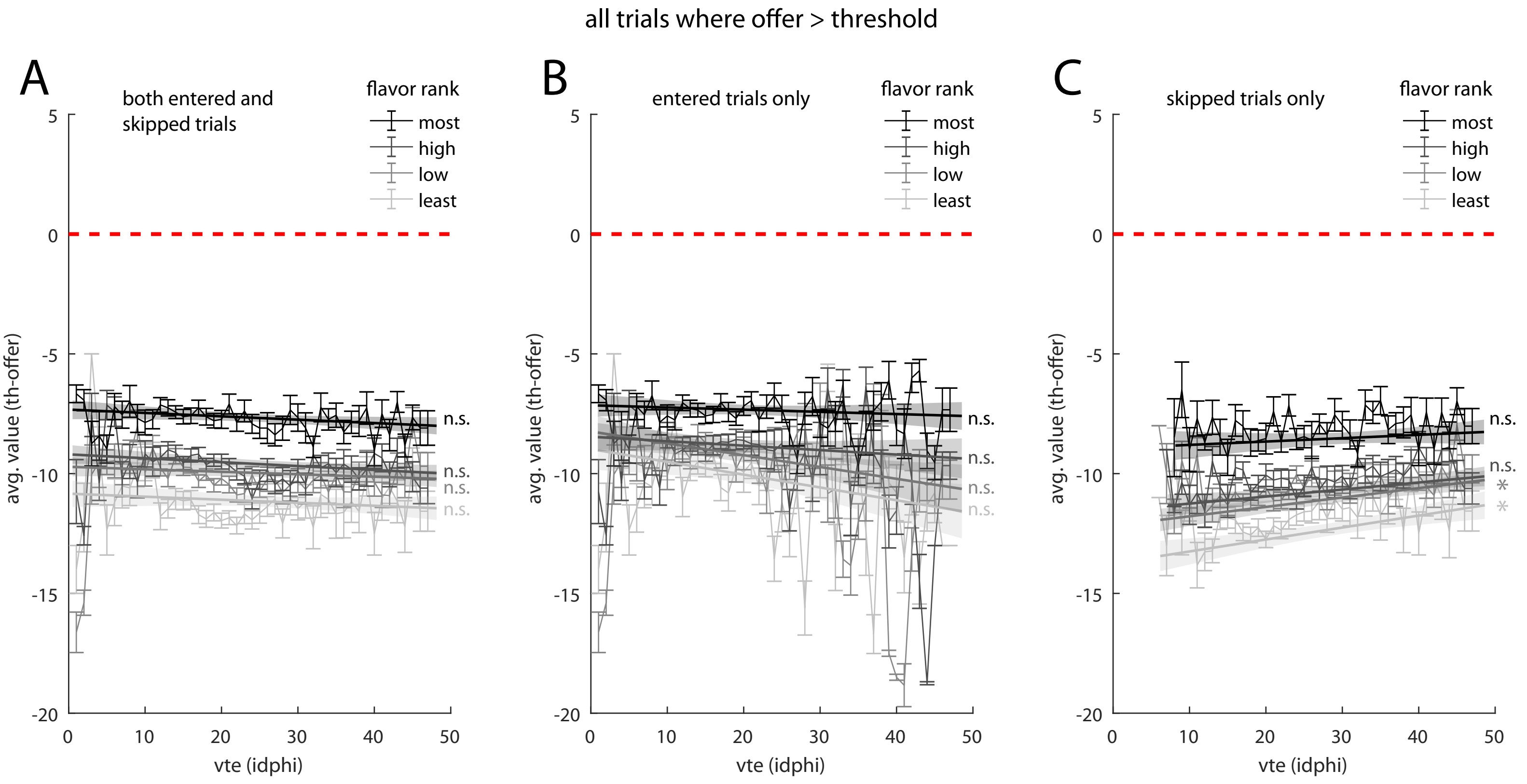

Supplementary Figure 5. Controlling for value as a function of VTE. The average value of offers encountered as a function of VTE measured on that trial are plotted split by restaurant ranking as well as decision outcome on that trial (A: skips and enters grouped together, B: enters only, C: skips only). Data presented here are derived from trials where offer > wait zone thresholds, representing bad deal trials. Thus, average values for all offers plotted here are <0 (horizontal dashed red line). As a function of VTE, offer value could explain some but not all changes in VTE. Correlation significance controlling for 12 multiple comparisons, Bonferroni corrected alpha level 0.05, *P<0.004, not significant (n.s.) P>0.004. Error bars. ± 1 SEM. Shaded error region displays 95% CI. N=31.

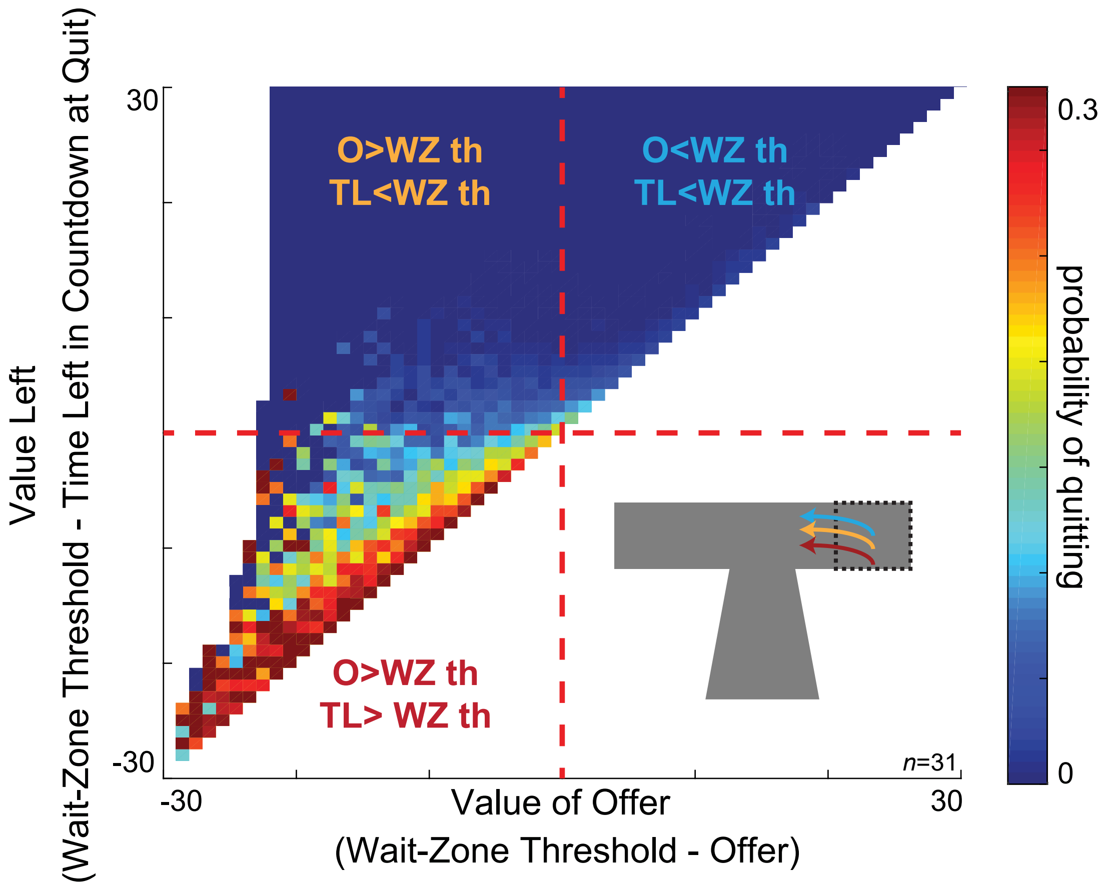

Supplementary Figure 6. Economic efficiency of quit events in the wait-zone. Accepted offers greater than wait-zone threshold are negatively valued offers (wait-zone threshold greater than offer). This is separated on the x-axis to the left of the vertical dashed red line indicating zero value (offers at wait-zone threshold). Additionally, the time remaining in the countdown at the time of quit was measured and value left was calculated by subtracting wait-zone threshold minus time left. Thus, negative value left in the countdown at quit is separated on the y-axis below the horizontal dashed red line indicating zero value left (time left in countdown at quit at wait-zone threshold). Majority of quits took place in the lower left quadrant (summarized in Fig.2L), indicating that the majority of quits occurred after mice had taken offers greater than their typical threshold (i.e. economically unfavorable), and the quit was a form of self-correction.

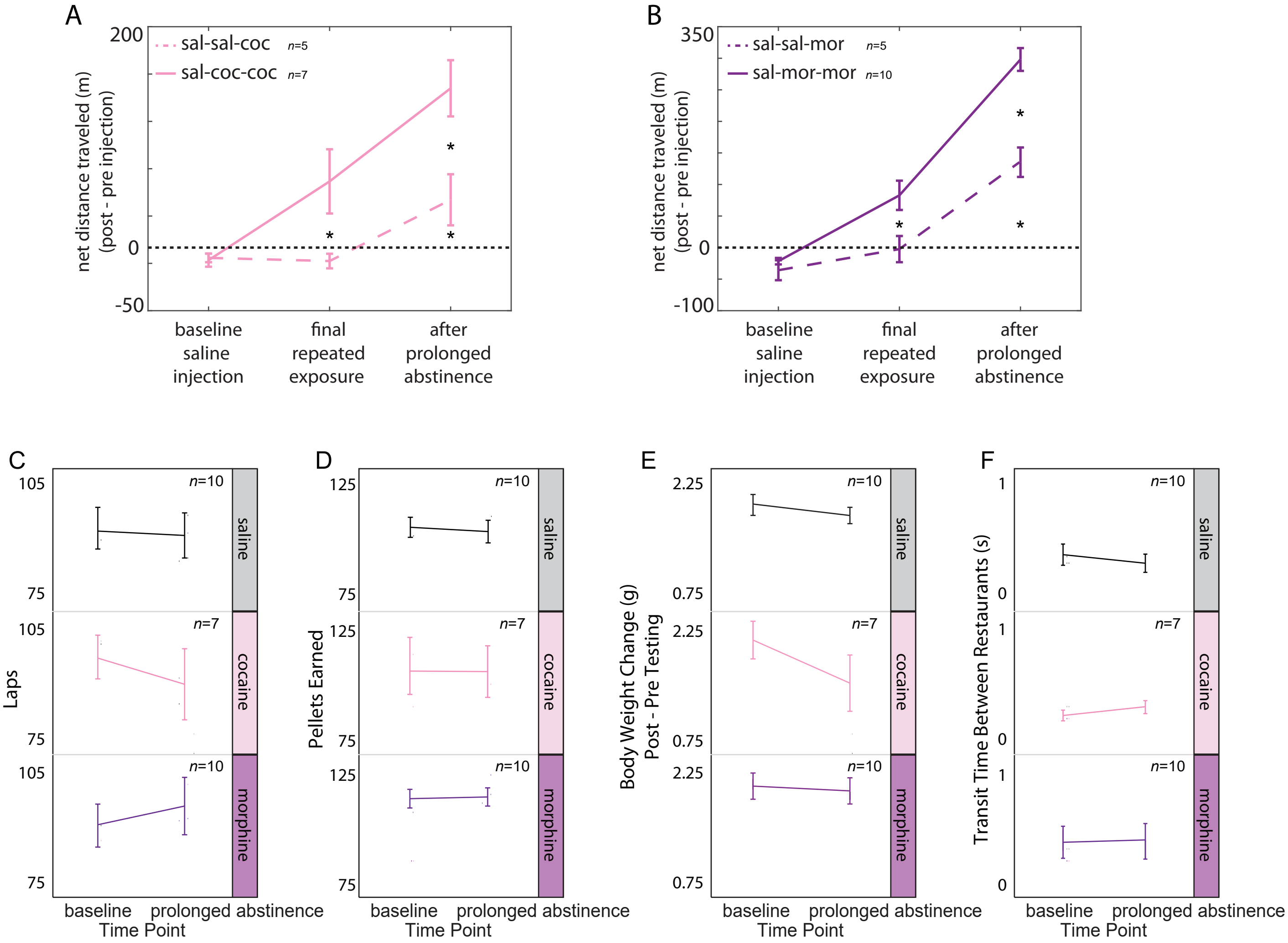

Supplementary Figure 7. Psychomotor sensitization and controlling for non-specific drug effects. (A-B) Locomotor activity immediately (measured in meters, m) before and after injections were measured in cocaine- (A) and morphine- (B) treated animals. All animals were injected with saline initially, then mice in the drug groups received repeated respective drug injections while control mice received repeated saline injections (locomotor response to final injection shown). Lastly, following 14 days of prolonged abstinence, enhanced psychomotor sensitization is expressed in mice with a history of drug use, not first-time drug-exposure in saline pre-treated mice. (Friedman, $P<0.05$, post-hoc Mann-Whitney locomotion comparisons between drug groups at time points, $*P<0.05$). (C-F) There were no lasting off-target effects on Restaurant Row Performance (C: Laps, D: Pellets Earned, E: Weight Change, F: Transit Speed). All four measures remained constant even after our drug and prolonged abstinence manipulation, implying that these off-target effects did not drive decision-making changes (Friedman, $P>0.05$). Error bars. ± 1 SEM. N per group listed on respective plots.

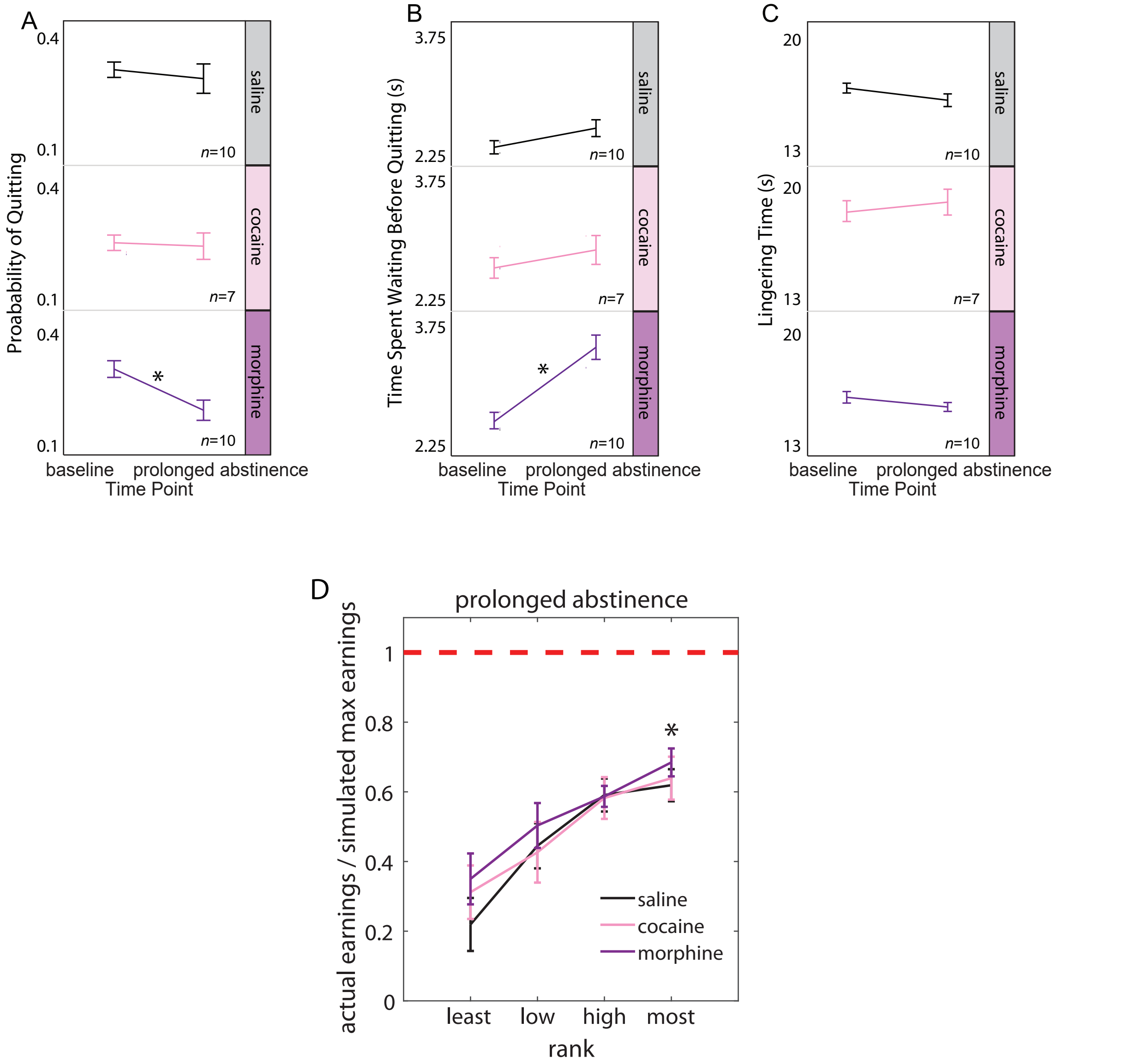

Supplementary Figure 8. Effects of prolonged abstinence on additional decision-making metrics. (A) Probability of quitting accepted offers in the wait-zone. (Friedman, morphine *P<0.05, saline/cocaine P>0.05) (B) Amount of time invested in the wait-zone before quitting. (Friedman, morphine *P<0.05, saline/cocaine P>0.05) (C) Amount of time spent consuming and lingering at the reward-site post-earning. (Friedman, all groups P>0.05) (D) Degree of optimal earnings. Sub-optimality was calculated by simulating Restaurant Row sessions and number of max potential earnable pellets using individual thresholds and running speeds, but removing wasteful behaviors (i.e., no quits, no excess time deliberating in offer-zone nor lingering post-earning beyond the minimum showed by the animal). Horizontal dashed red line indicated optimal performance as determined by simulations. (Sign test, P<0.05, all ranks below 1. Kruskal-Wallis-Dunn tests, most-preferred vs. least-preferred *P<0.05). Error bars. ± 1 SEM. N per group listed on respective plots.

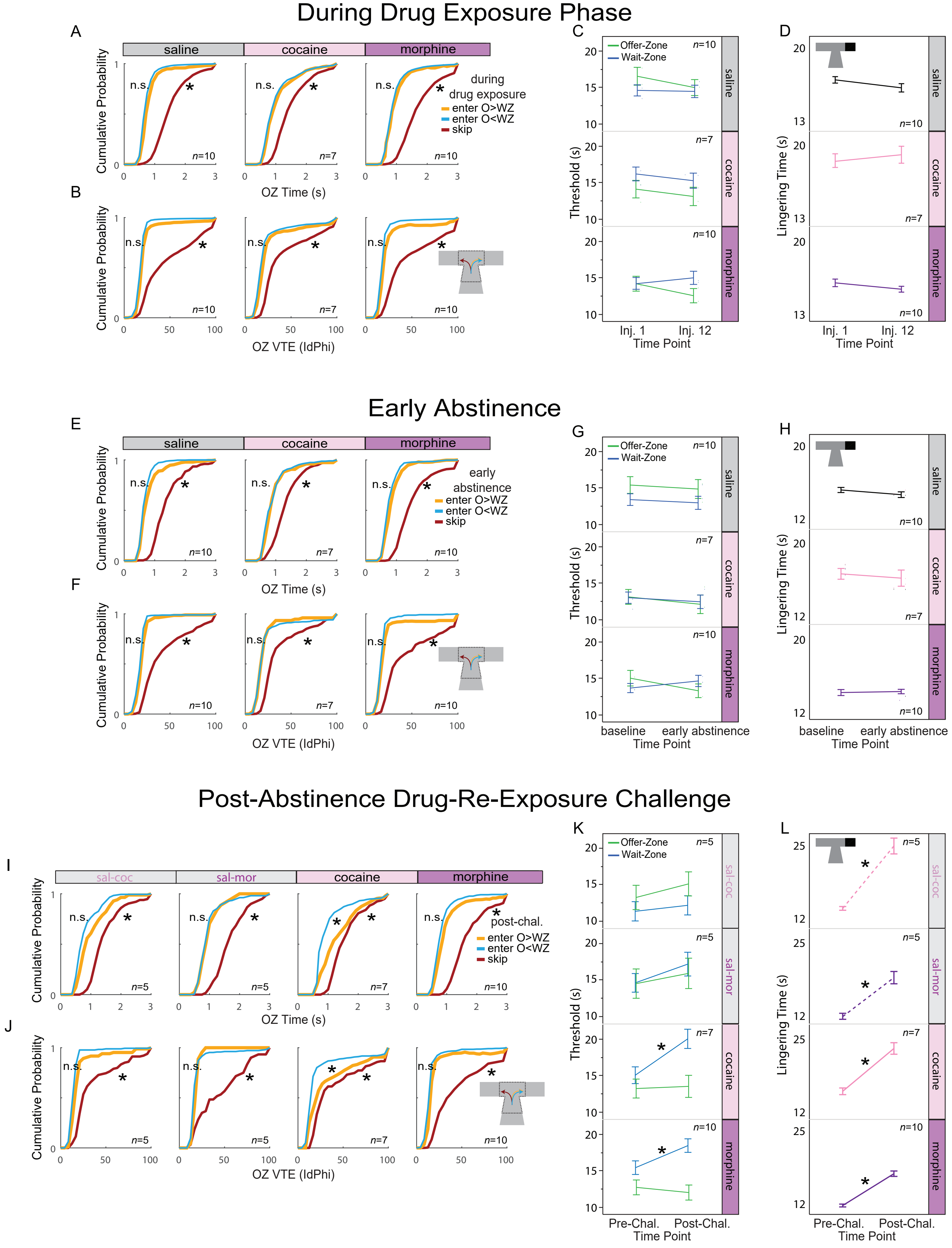
During Drug Exposure Phase
A
saline
cocaine
morphine
Cumulative Probability
n.s.
*
n=10
n=7
n=10
during drug exposure
enter O>WZ
enter O<WZ
skip
OZ Time (s)
B
OZ VTE (IdPhi)
C
Offer-Zone
Wait-Zone
Threshold (s)
Inj. 1
Inj. 12
Time Point
D
Lingering Time (s)
Early Abstinence
E
early abstinence
F
G
baseline
early abstinence
H
Post-Abstinence Drug-Re-Exposure Challenge
I
sal-coc
sal-mor
post-chal.
n=5
J
K
Pre-Chal.
Post-Chal.
L

Supplementary Figure 9. Secondary drug-related timepoints (cyan timepoints 1-3). Outside of the primary timepoints of comparison in this study (yellow timepoints in Fig.1A), we also examined behavior of multiple valuation parameters (offer-zone deliberations, offer- and wait-zone thresholds, and post-earn lingering) during the drug exposure phase (A-D), during early abstinence (E-H), and after the drug-re-exposure challenge intended to assess incubation of psychomotor sensitization (I-L) where mice were either re-exposed to the same drug previously administered or saline-pre-treated mice received either cocaine or morphine for the first time. (A-B) Cumulative probability distributions of offer-zone time (A) and VTE (B) for skips as well enters split by offer value separated by drug-treatment conditions collapsed across the drug exposure sequence. Both types of enter decisions were rapid compared to skip decisions (KS tests, *$P<0.05$) and indistinguishable from each other (KS tests, not significant, n.s., $P>0.05$) in all three drug conditions, similar to baseline findings. (C) Friedman tests revealed no changes in offer-zone and wait-zone thresholds across first and last injection of the repeated drug exposure sequence separated by drug-treatment conditions ($P>0.05$). (D) Similarly, no changes were found in time spent lingering at the reward site after earning across first and last injection of the repeated drug exposure sequence ($P>0.05$). (E-H) During early abstinence, no changes from baseline were observed in any of the valuation parameters. (E-F) Offer-zone time (E) and VTE (F) for enter decisions were both faster than skips (KS tests, *$P<0.05$) and indistinguishable from each other (KS tests, not significant, n.s., $P>0.05$) in all three drug conditions, similar to baseline findings. (G-H) Offer-zone and wait-zone thresholds (G) and lingering behavior (H) did not change over time (Friedman, $P>0.05$). (I-L) After the drug-re-exposure challenge, although cocaine-treated animals still displayed their main effect (following prolonged abstinence) of increase deliberation time (I) and VTE (J) for offers above wait-zone thresholds, no further changes were seen in any drug condition (KS tests, *$P<0.05$, not significant, n.s. $P>0.05$). (K) Only animals with a history of repeated drug exposure (both cocaine and morphine pre-treated groups) displayed increased wait-zone thresholds in response to an acute drug-re-exposure challenge while first-time-exposed mice did not (Friedman, *$P<0.05$). (L) All mice displayed an increase in lingering behavior following an acute drug challenge (Friedman, *$P<0.05$). Error bars. ± 1 SEM. N per group listed on respective plots.

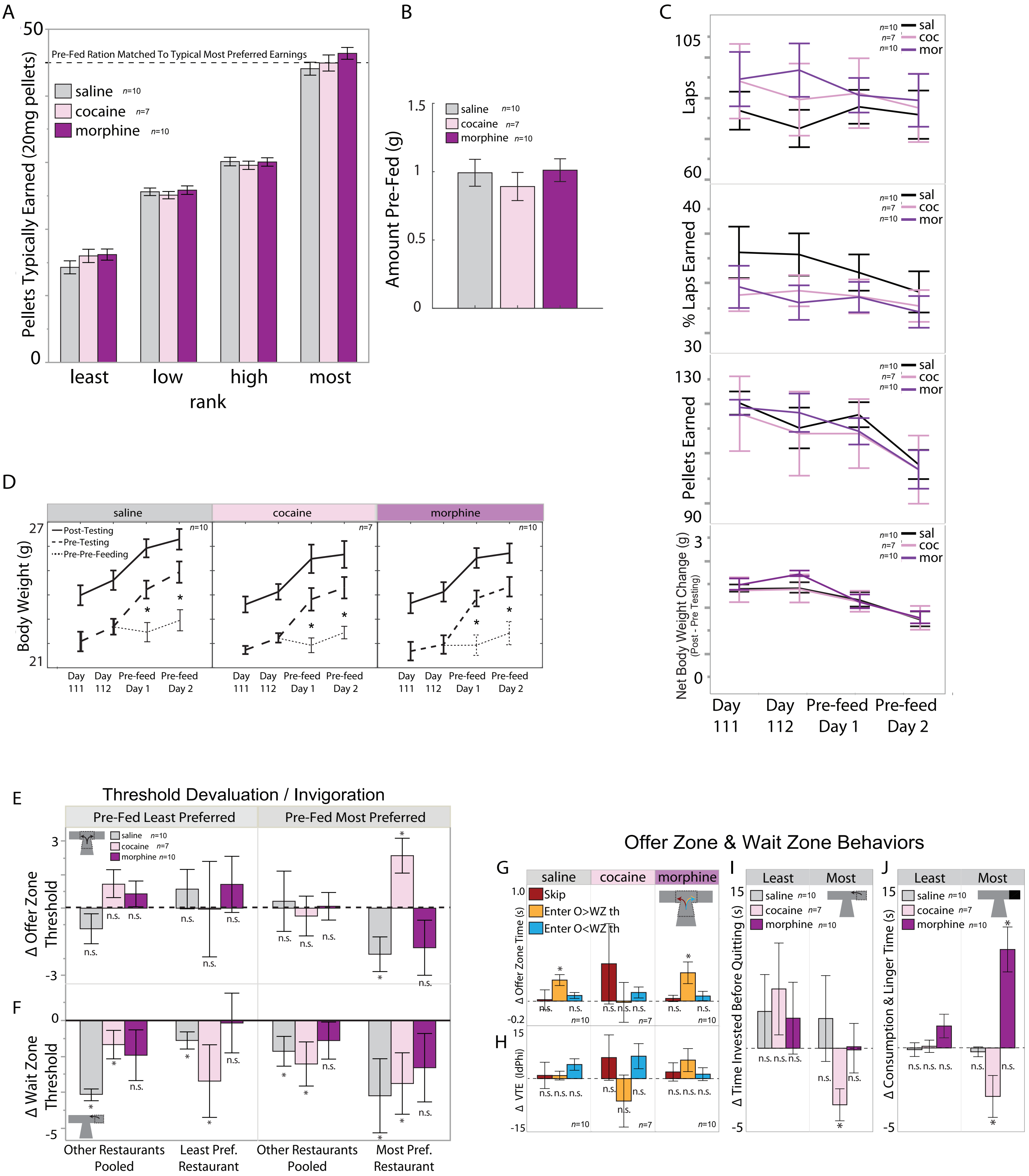
A
Pre-Fed Ration Matched To Typical Most Preferred Earnings
saline n=10
cocaine n=7
morphine n=10
Pellets Typically Earned (20mg pellets)
least
low
high
most
rank
B
Amount Pre-Fed (g)
C
Laps
% Laps Earned
Pellets Earned
Net Body Weight Change (g) (Post - Pre Testing)
sal
coc
mor
Day 111
Day 112
Pre-feed Day 1
Pre-feed Day 2
D
saline
cocaine
morphine
Post-Testing
Pre-Testing
Pre-Pre-Feeding
Body Weight (g)
E
Threshold Devaluation / Invigoration
Pre-Fed Least Preferred
Pre-Fed Most Preferred
Δ Offer Zone Threshold
F
Δ Wait Zone Threshold
Other Restaurants Pooled
Least Pref. Restaurant
Most Pref. Restaurant
Offer Zone & Wait Zone Behaviors
G
Skip
Enter O>WZ th
Enter O<WZ th
Δ Offer Zone Time (s)
H
Δ VTE (IdPhi)
I
Least
Most
Δ Time Invested Before Quitting (s)
J
Δ Consumption & Linger Time (s)

Supplementary Figure 10. Pre-feeding probe session (cyan timepoint 4). (A-B) Average number of pellets typically earned in each ranked restaurant. Dashed horizontal black line indicates the approximate number of pellets earned in a single session in most-preferred restaurants (A, ~45 pellets, 20mg each). This number was used to determine how much to pre-feed mice before devaluation probe sessions with the intention to partially satiate mice while preserving motivation to run the task following pre-feeding (B, ~0.9g). The same number was also used in both pre-feeding probe sessions regardless if pre-feeding with either the most- or least-preferred flavor. (C) Pre-feeding had no effect on laps run, % laps earned, pellets earned, or net-body-weight-change comparing pre-testing weights to post-testing weights (Friedman, $P>0.05$). (D) Pre-feeding however did increase body-weight when comparing pre-pre-feeding weights to post-pre-feeding weights measured before Restaurant Row testing (Friedman, *$P<0.05$). Pre-feeding plus additional weight gained during Restaurant Row did not significantly change starting pre-pre-feeding weight on the second probe session (Friedman, $P>0.05$). (E-J) We measured changes in behavior of multiple valuation parameters (offer- and wait-zone thresholds, offer-zone deliberations, wait-zone quits, and post-earn lingering) by calculating changes relative to 5d of average behavior preceding the first pre-feeding session (Sign tests, *$P<0.05$, not significant, n.s., $P>0.05$). (F) Mice with a history of repeated saline or cocaine exposure showed decreased wait-zone thresholds in all restaurants in response to pre-feeding regardless of the identity of the pre-fed flavor. Morphine pre-treated mice displayed no changes in wait-zone thresholds. (E) In the offer-zone, thresholds of only the most-preferred flavor only when pre-fed that flavor decreased in saline mice, increased in cocaine mice, and did not change in morphine mice. No other offer-zone thresholds changed in all mice. (G-H) Only saline- and morphine-mice showed increased offer-zone reaction times when accepting offers above wait-zone threshold (G) in the most-preferred restaurant when pre-fed that flavor, however these changes were not accompanied with changes in vicarious trial and error (VTE) behavior (H). (I-J) Cocaine-mice showed a decrease in time invested before quitting (I) and a decrease in time spent lingering (J) in the most-preferred restaurant when pre-fed that flavor while morphine mice only showed an increase in lingering time. Saline mice displayed no changes in wait-zone quit time or post-earn lingering after pre-feeding. Error bars. ± 1 SEM. N per group listed on respective plots.

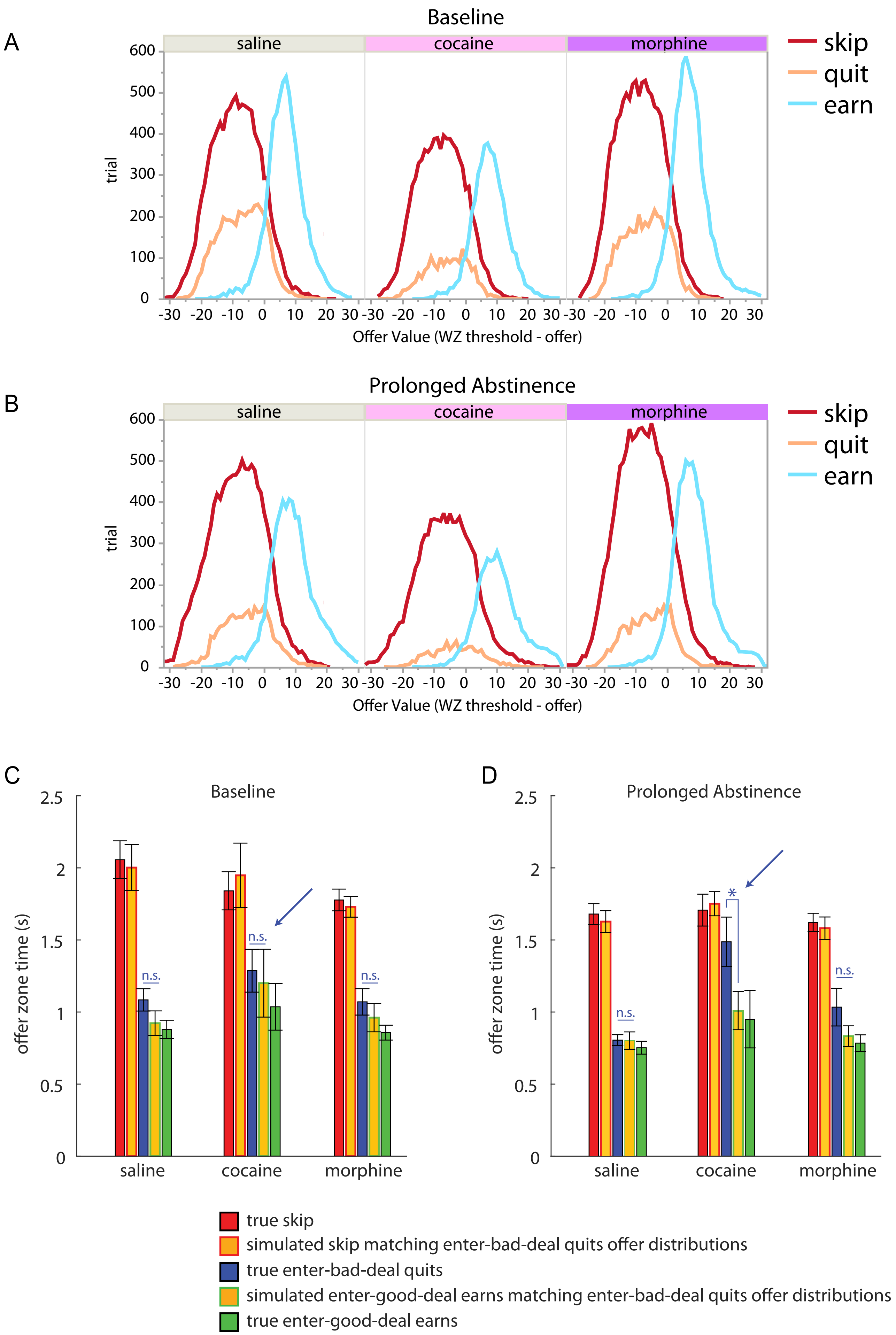
A
Baseline
saline
cocaine
morphine
skip
quit
earn
trial
600
500
400
300
200
100
0
-30 -20 -10 0 10 20 30
Offer Value (WZ threshold - offer)
B
Prolonged Abstinence
C
Baseline
offer zone time (s)
2.5
2
1.5
1
0.5
0
n.s.
saline
cocaine
morphine
D
Prolonged Abstinence
*
true skip
simulated skip matching enter-bad-deal quits offer distributions
true enter-bad-deal quits
simulated enter-good-deal earns matching enter-bad-deal quits offer distributions
true enter-good-deal earns

Supplementary Figure 11. Controlling for offer distribution differences in decision outcomes. (A-B) Decision types sorted by trial outcome as a function of offer value (wait zone threshold minus offer) across all animals split by treatment group at baseline (A) or prolonged abstinence (B) intended to illustrate different decision outcomes occur on trials with very different offer distributions, particularly the two types of enter decisions (enter then earn, enter then quit). (C-D) We ran single trial simulation analyses during baseline (C) or prolonged abstinence (D) to control for unequal distributions of offers based on trial type (skipping bad deal, entering bad deal then quitting, or entering good deal) that could confound interpretations of offer zone behaviors when making initial enter or skip decisions. We generated simulated shuffled data sets of both skipping a bad deal and entering a good deal then earning matching the same trial-by-trial distributions of offer lengths as those subsets of trials where mice entered a bad deal then quit. That is, simulations were performed by using the offer length distributions that belong to the enter-bad-deal scenario and then averaging only those offer-zone reaction times that matched this offer distribution where the outcomes were instead skips (for the skip simulation) or enter-good-deals (for the enter simulation). We found that after running these analyses on baseline days 66-70, we do not see any significant differences in any treatment group between our conditions of interest (how mice deliberated before accepting bad deals in the offer-zone), comparing entering bad offers that leads to quits to the shuffled control entering that leads to earns simulated to match offer distribution of entering bad deals before quitting, ($P>0.05$). This comparison of interest does change even when matched against simulated shuffled data sets only in the cocaine group after prolonged abstinence (*$P<0.05$). Thus, offer-zone behavior when entering-bad-deals looks like entering-good-deals (both are rapid snap judgments even if the former is a mistake) for all mice at both time points except the cocaine-treated animals at the prolonged abstinence time point. Error bars. ± 1 SEM. N per group listed on respective plots.

Supplementary References

1. Redish, A. D. Vicarious trial and error. *Nat. Rev. Neurosci.* 17, 147–59 (2016).
2. Thomas, M. J., Beurrier, C., Bonci, A., Malenka, R. C. Long-term depression in the nucleus accumbens: A neural correlate of behavioral sensitization to cocaine. *Nature Neuroscience* 4:1217-1223. (2001).
3. Hearing, M. *et al.* Reversal of morphine-induced cell-type–specific synaptic plasticity in the nucleus accumbens shell blocks reinstatement. *Proceedings of the National Academy of Sciences* 113, 757–762 (2016).
4. Robinson, T. E., Berridge, K. C. The psychology and neurobiology of addiction: an incentive-sensitization view. *Addiction* 95(2), S91–S117. (2000).
5. Camchong, J. *et al.* Changes in resting functional connectivity during abstinence in stimulant use disorder: a preliminary comparison of relapsers and abstainers. *Drug Alcohol Depend* 139, 145–51 (2014).
6. Colwill, R. M., Rescorla, R. A.. Effect of reinforcer devaluation on discriminative control of instrumental behavior. *Journal of experimental psychology. Animal behavior processes* 16, 40–7 (1990).
7. Balleine, B. W., Dickinson, A. The role of incentive learning in instrumental outcome revaluation by sensory-specific satiety. *Animal Learning & Behavior* 26, 46–59 (1998).
8. Wassum, K. M., Cely, I. C., Maidment, N. T., Balleine, B. W. Disruption of endogenous opioid activity during instrumental learning enhances habit acquisition. *Neuroscience* 163, 770–80 (2009).
9. Gremel, C. *et al.* Endocannabinoid Modulation of Orbitostriatal Circuits Gates Habit Formation. *Neuron* 90, 1312–1324 (2016).
10. Gourley, S. L., Zimmermann, K. S., Allen, A. G., Taylor, J. R. The Medial Orbitofrontal Cortex Regulates Sensitivity to Outcome Value. *J. Neurosci.* 36, 4600–13 (2016).
11. Johnson, A., Redish, A. D. Neural ensembles in CA3 transiently encode paths forward of the animal at a decision point. *Journal of Neuroscience* 27(45):12176-12189 (2007).
12. Gupta, A. S., M.A.A. van der Meer, Touretzky, D. S., Redish, A. D. Segmentation of spatial experience by hippocampal theta sequences. *Nature Neuroscience* 15:1032-1039. (2012).
13. Papale, A. E., Zielinski, M. C., Frank, L. M., Jadhav, S. P., Redish, A. D. Interplay between Hippocampal Sharp-Wave-Ripple Events and Vicarious Trial and Error Behaviors in Decision Making. *Neuron* 92, 975–982 (2016).
14. Wikenheiser, A. M., Redish, A. D.. Hippocampal theta sequences reflect current goals. *Nature neuroscience* 18:289-294 (2015).
15. Johnson, A., van der Meer, M., Redish, A. D. Integrating hippocampus and striatum in decision-making. *Current opinion in neurobiology* 17, 692–7 (2007).
16. Papale, A. E., Zielinski, M. C., Frank, L. M., Redish, A. D.. Interplay between hippocampal sharp-wave-ripple events and vicarious trial and error behaviors in decision making. *Neuron* 92(5):975-982 (2016).
17. Van der Meer, M. A. A., Redish, A. D. Covert expectation-of-reward in rat ventral striatum at decision points. *Frontiers in Integrative Neuroscience* 3(1):1-15 (2009).
18. Van der Meer, M., Redish, A. D. Low and high gamma oscillations in rat ventral striatum have distinct relationships to behavior, reward, and spiking activity on a learned spatial decision task. *Frontiers in integrative neuroscience* 3:9 (2009).

19. Steiner, A., Redish, A. D. The road not taken: neural correlates of decision making in orbitofrontal cortex. *Frontiers in neuroscience* 6, 131 (2012).
20. Stott, J., Redish. A. D. Functional difference in information processing between orbitofrontal cortex and ventral striatum during decision-making behaviour. *Philosophical transactions of the Royal Society of London. Series B, Biological sciences* 369, (2014).
21. Van der Meer, M., Johnson, A., Schmitzer-Torbert, N. C., Redish, A. D. Triple dissociation of information processing in dorsal striatum, ventral striatum, and hippocampus on a learned spatial decision task. *Neuron* 67:25-32 (2010).
22. Smith, K. S., Greybiel, A. M.. A dual operator view of habitual behavior reflecting cortical and striatal dynamics. *Neuron* 79(2):361-374 (2013).
23. Regier, P. S., Amemiya, S., Redish, A. D. Hippocampus and subregions of the dorsal striatum respond differently to a behavioral strategy change on a spatial navigation task. *J Neurophys* 114(3):1399-1416 (2015).
24. Schmidt, B., Papale, A., Redish, A. D., Markus, E. Conflict between place and response navigation strategies: effects on vicarious trial and error (VTE) behaviors. *Learning & memory* 20, 130–8 (2013).
25. Amemiya, S., Redish, A. D. Manipulating Decisiveness in Decision Making: Effects of Clonidine on Hippocampal Search Strategies. *J. Neurosci.* 36, 814–27 (2016).
26. Gardner, R. S., *et al*. A secondary working memory challenge preserves primary place strategies despite overtraining. *Learning & Memory* 20(11), 648-656 (2013).
27. Hasz, B. M., Redish, A. D. A two-step decision-task for rats reveals behavioral correlates of model-based and model-free decisions. *SFN*. (2016).
28. Stephens, D., Krebs, J. Foraging Theory. Princeton Univ Press, Princeton (1987).
29. Verstraete, A. G. Detection Times of Drugs of Abuse in Blood, Urine, and Oral Fluid. *The Drug Monit* 26(2), 200-205 (2004).
30. Robinson, T., Berridge, K. Addiction. *Annual review of psychology* 54, 25–53 (2003).
31. Wolf, M. E. Synaptic mechanisms underlying persistent cocaine craving. *Nat. Rev. Neurosci.* 17, 351–65 (2016).
32. Kourrich, S., Rothwell, P., Klug, J., Thomas, M. J. Cocaine Experience Controls Bidirectional Synaptic Plasticity in the Nucleus Accumbens. *The Journal of Neuroscience* 27, 7921–8 (2007).
33. Kourrich, S., Klug, J. R., Mayford, M., Thomas, M. J. AMPAR-independent effect of striatal αCaMKII promotes the sensitization of cocaine reward. *J. Neurosci.* 32, 6578–86 (2012).
34. Robinson, T. E., Berridge, K. C. The neural basis of drug craving: an incentive-sensitization theory of addiction. *Brain research. Brain research reviews* 18, 247–91 (1993).
35. Robinson, T. E., Berridge, K. C. The incentive sensitization theory of addiction: some current issues. *Philosophical Transactions of the Royal Society B* 363: 3137:3146. (2008).
36. Rothwell, P. E., Kourrich, S., Thomas, M. J. Synaptic adaptations in the nucleus accumbens caused by experiences linked to relapse. *Biol. Psychiatry* 69, 1124–6 (2011).
